# Female Genital Mutilation/Cutting: sharing data and experiences to accelerate eradication and improve care: part 2: Geneva, Switzerland. March 13-14, 2017

**DOI:** 10.1186/s12978-017-0362-x

**Published:** 2017-09-15

**Authors:** Fabienne Richard, Wisal Ahmed, Nikki Denholm, Angela Dawson, Nesrin Varol, Birgitta Essén, Sara Johnsdotter, Paul Bukuluki, Wisal Ahmed, Al Gasseer H. Naeema, Dalya eltayeb, Bettina Shell-Duncan, Caroline Njue, Jacinta Muteshi, Clotilde Lamy, Pascale Neyrinck, Fabienne Richard, Peter Verduyckt, Sophie Alexander, Samuel Kimani, Tammary Esho, Violet Kimani, Christine Kigondu, Joseph Karanja, Jaldesa Guyo, Moustapha Touré, Yacin Gackou Guindo, Dramane Samaké, Ladji Camara, Youssouf Traoré, Alassane A. Traoré, Alou Samaké, Crista E. Johnson-Agbakwu, Malin Jordal, Elena Jirovsky, Samantha Wu, Kevin Fitzgerald, Ranit Mishori, Rebecca Reingold, Edna Adan Ismail, Lale Say, Marion Uebelhart, Michel Boulvain, Patrick Dallenbäch, Olivier Irion, Patrick Petignat, Jasmine Abdulcadir, Patrizia Farina, Els Leye, Livia Ortensi, Claudia Pecorella, Lindsey Novak, Jasmine Abdulcadir, Béatrice Cuzin, Florence Brunel Delmas, Albertine Papingui, Dina Bader, Anna Wahlberg, Sara Johnsdotter, Katarina Ekholm Selling, Carina Källestål, Birgitta Essén, Abdalla Hisham Hussein Imam Ibraheim, Nasr A. M. Elawad, Wisal Ahmed, Al Gasseer, H. Naeema, Elamin Maison, Hiba Hussein, Altayyeb Mohammed Albagir, Paul Bukuluki, Mohamed Tawfig Albirair, Sarah A. Salam Salih, Wisal Ahmed, Al Gasseer, H. Naeema, Elamin Maison, Hiba Hussein, Altayyeb Mohammed Albagir, Mohamed Tawfig Albirair, Paul Bukuluki, Angela Dawson, Nesrin Varol, Tammary Esho, Samuel Kimani, Violet Kimani, Samuel Muniu, Christine Kigondu, Isaac Nyamongo, Jaldesa Guyo, Patrick Ndavi, Rebecca Reingold, Ranit Mishori, Kevin Fitzgerald, Samantha Wu, Holly Hedley, Rachel Kuenzi, Laura Malavé-Seda, Camille Clare, Jacqueline Greenfield, Praise Augustus, Nneamaka Ukatu, Eugene Manu, Brian Altonen, Martin Caillet, Fabienne Richard, Pierre Foldès, Béatrice Cuzin, Florence Brunel Delmas, Albertine Papingui, Sophie Wylomanski, Mathilde Vital, Sophie De Visme, Stéphanie Dugast, Matthieu Hanf, Norbert Winer, Sara Johnsdotter, Birgitta Essén, Amr Seifeldin, Ranit Mishori, Kevin Fitzgerald, Rebecca Reingold, Samantha Wu, Michela Villani, Sara Johnsdotter, Birgitta Essén, Rebecca Seinfeld, Brian Earp, S. Cappon, C. L’Ecluse, E. Clays, I. Tency, E. Leye, R. E. Johansen, C. M. Ouédraogo, S. Madzou, A. Simporé, V. Combaud, A. Ouattara, F. Millogo, A. Ouédraogo, S. Kiemtore, H. Zamane, Y. A. Sawadogo, P. Kaien, B. Dramé, B. Thieba, J. Lankoandé, P. Descamps, L. Catania, R. Mastrullo, A. Caselli, R. Cecere, O. Abdulcadir, J. Abdulcadir, Sonja Vogt, Charles Efferson, S. O’Neill, D. Dubour, S. Florquin, M. Bos, S. Zewolde, F. Richard, N. Varol, A. Dawson, S. Turkmani, J. J. Hall, S. Nanayakkara, G. Jenkins, C. S. Homer, K. McGeechan, M. Vital, S. de Visme, M. Hanf, H. J. Philippe, N. Winer, S. Wylomanski, C. Johnson-Agbakwu, N. Warren, A. Macfarlane, W. Dorkenoo, I. L. Lien, J. H. Schultz

**Affiliations:** 1 CeMAViE, University Hospital of St-Pierre, Brussels, Belgium; 2 Institute of Tropical Medicine, Antwerp, Belgium; 3 GAMS, Brussels, Belgium; 4 Federal Ministry of Health (Maternal Child Health Directorate and Human Resource Directorate -CPD), River Nile and Northern State Ministry of Health and World Health Organization, Khartoum, Sudan; 5 New Zealand National FGM Education Programme, New Zealand Ministry of Health Provider, Auckland, New Zealand; 6 The Australian Centre for Public and Population Health Research, Faculty of Health, The University of Technology Sydney, Sydney, Australia; 7 Sydney Medical School, University of Sydney, Sydney, Australia; 8 Uppsala University, Uppsala, Sweden; 9 Malmö University, Malmö, Sweden; 10 World Health Organization, Khartoum, Sudan; 11 Federal Ministry of Health (Maternal Child Health Directorate and Human Resource Directorate -CPD), Khartoum, Sudan; 12 University of Washington, Seattle, Washington USA; 13 Population Council, Naironi, Kenya; 14 Iris Sud Hospitals, Brussels, Belgium; 15 GAMS Belgium, Brussels, Belgium; 16 Brussels Health and Social Observatory, Brussels, Belgium; 17 Université Libre of Brussels, Brussels, Belgium; 18 University of Nairobi, Nairobi, Kenya; 19 Africa Coordinating Centre for Abandonment of Female Genital Mutilation/Cutting, Nairobi, Kenya; 20 Technical University of Kenya, Nairobi, Kenya; 21 Mali University Hospital, Bamako, Mali; 22 National program against female circumcision, Bamako, Mali; 23 Marie Stoppes International, Bamako, Mali; 24 Ministry of Environment, Bamako, Mali; 25 Gabbriel Touré University Hospitals, Bamako, Mali; 26 Referral Health Center. Commune 6, Bamako, Mali; 27 Founding Director, Refugee Women’s Health Clinic, Maricopa Integrated Health System, Obstetrics & Gynecology, Phoenix, Arizona USA; 28 Assistant Research Professor, Arizona State University, Southwest Interdisciplinary Research Center, Phoenix, Arizona USA; 29 Center for Gender Research, Uppsala, Sweden; 30 Medical University of Vienna, Center for Public Health, Department of General Practice and Family Medicine, Vienna, Austria; 31 Pellegrino Center for Clinical Bioethics. Georgetown University Medical Center, Washington DC, USA; 32 Department of Family Medicine, Georgetown University School of Medicine, MedStar Health – Family Medicine at Spring Valley, Washington DC, USA; 33 O’Neill Institute for National and Global Health Law, Georgetown University Law Center, Washington DC, USA; 34 Edna Adan Hospital, Hargeisa, Somaliland Somalia; 35 Department of Reproductive Health and Research, World Health Organization, Geneva, Switzerland; 36 Faculty of Medicine, University of Geneva, Geneva, Switzerland; 37 Department of Obstetrics and Gynecology, Geneva University Hospitals, Geneva, Switzerland; 38 Department of Sociology and Social research, University of Milan Bicocca, Milan, Italy; 39 International Centre for Reproductive Health, Ghent University, Ghent, Belgium; 40 Department of Law, University of Milan Bicocca, Milan, Italy; 41 University of Minnesota, Minneapolis, Minnesota USA; 42 Faculty of Medicine, University of Geneva, Department of Obstetrics and Gynecology, Geneva University Hospitals, Geneva, Switzerland; 43 GH E Herriot, Lyon, France; 44 GAMS, Lyon, France; 45 Institute of Social Sciences, University of Lausanne, Lausanne, Switzerland; 46 Swiss Forum for Migration and Population Studies, University of Neuchâtel, Neuchâtel, Switzerland; 47 Uppsala University, Uppsala, Sweden; 48 Malmö University, Malmö, Sweden; 49 Faculty of Medicine, Minia University, Minia, Egypt; 50 Sudan Obstetric and Gynecological Society, Khartoum, Sudan; 51 World Health Organization Khartoum, Khartoum, Sudan; 52 Public Health Institute, Khartoum, Sudan; 53 Sudan Association of Pediatricians, Khartoum, Sudan; 54 World Health Organization, Khartoum, Sudan; 55 Public Health Institute, Khartoum, Sudan; 56 The Australian Centre for Public and Population Health Research, Faculty of Health., University of Technology Sydney, Sydney, Australia; 57 Sydney Medical School, University of Sydney, Sydney, Australia; 58 African Coordinating Centre for Abandonment of FGM/C, University of Nairobi, Kenyatta National Hospital, P.O Box 19676-00202, Nairobi, Kenya; 59 Department of Community and Public Health, Technical University of Kenya, P.O Box 52426, Nairobi, Kenya; 60 School of Nursing sciences, Kenyatta National Hospital, P.O Box 19676-00202, Nairobi, Kenya; 61 School of Public Health, Kenyatta National Hospital, P.O Box 19676-00202, Nairobi, Kenya; 62 Department of Clinical Chemistry, University of Nairobi, Kenyatta National Hospital, P.O Box 19676-00202, Nairobi, Kenya; 63 Institute of Anthropology, gender and African studies, University of Nairobi, Nairobi, Kenya; 64 Department of Obstetrics and Gynaecology, University of Nairobi, Kenyatta National Hospital, P.O Box 19676-00202, Nairobi, Kenya; 65 O’Neill Institute for National and Global Health Law, Georgetown University Law Center, Washington DC, USA; 66 Department of Family Medicine, Georgetown University School of Medicine, MedStar Health – Family Medicine at Spring Valley, Washington DC, USA; 67 Pellegrino Center for Clinical Bioethics, Georgetown University Medical Center, Washington DC, USA; 68 Georgetown University Law Center, Washington DC, USA; 69 New York Medical College, New York, New York USA; 70 Lehman College, Bronx, New York USA; 71 New York City Health and Hospitals Corporation, New York, New York USA; 72 CeMAViE, University Saint Pierre hospital, Brussels, Belgium; 73 Institut en Santé Génésique, Saint Germain en Laye, Paris, France; 74 GH E Herriot, Lyon, France; 75 GAMS, Lyon, France; 76 Department of Gynecology and Obstetrics, Nantes University Hospital, Nantes, France; 77 INSERM CIC 1413, Nantes University Hospital, Nantes, France; 78 Malmö University, Malmö, Sweden; 79 Uppsala University, Uppsala, Sweden; 80 Urogynecology & pelvic reconstructive surgery unit, El Galaa teaching hospital, Cairo, Egypt; 81 Department of Family Medicine, Georgetown University School of Medicine, MedStar Health – Family Medicine at Spring Valley, Washington DC, USA; 82 Pellegrino Center for Clinical Bioethics, Georgetown University Medical Center, Washington DC, USA; 83 O’Neill Institute for National and Global Health Law, Georgetown University Law Center, Washington DC, USA; 84 University of Fribourg, Fribourg, Switzerland; 85 Malmö University, Malmö, Sweden; 86 Uppsala University, Uppsala, Sweden; 87 Visiting Research Fellow, Centre of the Body, Goldsmiths, University of London, London, United Kingdom; 88 Associate Director, Yale-Hastings Program in Ethics & Health Policy and Departments of Philosophy and Psychology, Yale University, New Heaven, Connecticut USA; 89 Faculty of Medicine, International Centre for Reproductive Health, Ghent University, Gent, Belgium; 90 Department Public Health, Ghent University, Gent, Belgium; 91 Midwifery Department, KAHO Sint Lieven, Sint Niklaas, Belgium; 92 Norwegian Center for Violence and Traumatic Stress Studies, Oslo, Norway; 93 Division of Gynecology and Obstetrics, CHU Yalgado Ouédraogo, Ouagadougou, Burkina Faso; 94 Pôle Femme-Mère-Enfant, CHU d’Angers, Angers, France; 95 Regional Referral Centre for the Treatment and Prevention of FGM, Health Promotion of Immigrant Woman, Department of Science for Woman and Child Health, University of Florence, Florence, Italy; 96 Faculty of Medicine, University of Geneva, Geneva, Switzerland; 97 Department of Obstetrics and Gynecology, Geneva University Hospitals, Geneva, Switzerland; 98 University of Zurich, Zurich, Switzerland; 99 Institute of Tropical Medicine, Antwerp, Antwerp, Belgium; 100 GAMS, Brussels, Belgium; 101 HIMILO Foundation, Amsterdam, The Netherlands; 102 FORWARD UK, London, UK; 103 Discipline of Obstetrics and Gynaecology, Sydney Medical School, University of Sydney, Sydney, NSW Australia; 104 Centre for Midwifery, Child and Family Health, Faculty of Health, University of Technology Sydney, Sydney, NSW Australia; 105 Centre for Clinical Epidemiology & Biostatistics, School of Medicine and Public Health, Faculty of Health and Medicine, University of Newcastle, Sydney, NSW Australia; 106 Department of Obstetrics and Gynaecology, Auburn Hospital, Sydney, NSW Australia; 107 Discipline of Obstetrics and Gynaecology, School of Medicine, Notre Dame University, Sydney, NSW Australia; 108 School of Public Health, University of Sydney, Sydney, NSW Australia; 109 Department of Gynecology and Obstetrics, Nantes University Hospital, Nantes, France; 110 INSERM CIC 1413, Nantes University Hospital, Nantes, France; 111 Department of Gynecology and Obstetrics, Nantes University Hospital, Nantes, France; 112 Refugee Women’s Health Clinic, Maricopa Integrated Health System, Obstetrics & Gynecology, Phoenix, Arizona USA; 113 Arizona State University, Southwest Interdisciplinary Research Center, Phoenix, Arizona USA; 114 Department Cmmunity Public Health Nursing. Johns Hopkins School of Nursing, Baltimore, Maryland USA; 115 City, University of London, London, UK; 116 Norwegian Centre for Violence and Traumatic Stress Studies, Oslo, Norway

## I. Healthcare professionals: training and curricula. Medicalization

### 1 Healthcare professionals training on FGM: challenges and opportunities: a commentary

#### Fabienne Richard^1,2,3^ (fabienne@gams.be)

##### ^1^CeMAViE, University Hospital of St-Pierre, Brussels, Belgium; ^2^Institute of Tropical Medicine, Antwerp, Belgium; ^3^GAMS, Brussels, Belgium

Health professionals have a critical role to play in the prevention and management of FGM. However, several KAP (Knowledge, Attitudes and Practices) studies conducted in high and low income countries have shown a lack of knowledge on WHO classification, diagnosis and management of FGM. Although several countries have developed FGM guidelines for professionals and have voted specific laws against the practice, the studies showed that these measures are not sufficient and that educational activities are needed to implement existing guidelines. Integration of the thematic in the curriculum of health professionals is a longstanding recommendation, but few countries have done it. Evidence of best practices in educational programs is lacking; a recent review only found two studies meeting the study selection criteria. There is a need for high-quality research on educational strategies using common indicators in order to allow comparisons between country programs. Professionals need operational tools: case studies, videos, and pictures. New technology is an opportunity: E-learning tools and visual tools to identify different types of FGM could make the difference. The KAP studies have also shown the ambivalence of health staff who are caring for women affected by FGM but may also perform FGM in some contexts (medicalization). There is a need for the integration of a discussion on ethics and the role of professionals in prevention in training programs. The last point highlighted by the KAP studies is the lack of knowledge of professionals on the cultural context and on psychological and sexual consequences of FGM (and its management). This should be the new focus of training modules on FGM, established with the active participation of psychologists, sexologists, and peer educators from the community.

### 2 Quality improvement method to optimize Female Genital Mutilation (FGM): protocol for an in-service training package targeting community midwives in Northern and River Nile states in Sudan

#### Wisal Ahmed (wahmed@who.int)

##### Federal Ministry of Health (Maternal Child Health Directorate and Human Resource Directorate -CPD), River Nile and Northern State Ministry of Health and World Health Organization, Khartoum, Sudan

**Introduction**

The implementation of methods to increase knowledge and skills among health workers adapted to the contextual settings and its effectiveness on medicalization is not much studied. Sudan has a high FGM prevalence with increasing medicalization rates, mostly performed by trained midwives. This method paper details a four-step protocol for an analysis and improvement intervention designed to develop a tailored in-service training package to community midwives in two states in Sudan is currently underway.

**Methods**

This study employs a two-arm, longitudinal randomized trial design targeting 1,000 community midwives. The Consolidated Framework for Implementation Research (CFIR) will be used to guide collection and analysis of qualitative data on implementation process. Plan Do Study Act cycles will be used to rapidly and regularly assess knowledge, attitudes and skills sets to iteratively improve on the intervention.

**Results**

This study is expected to provide guidance for improving knowledge and skill sets for community midwives and evaluating the effectiveness of this intervention on type of interventions on FGM de-medicalization.

**Conclusion**

Implementation research is a much-needed area to study the delivery of strategic approaches to accelerate de-medicalization.

### 3 The New Zealand (NZ) national FGM education program: case study of a successful training and prevention model for NZ healthcare professionals

#### Nikki Denholm (nikki@exposure.org)

##### New Zealand National FGM Education Programme, New Zealand Ministry of Health Provider, Auckland, New Zealand

**Introduction**

The New Zealand Female Genital Mutilation (FGM) Education Programme (NZFGMP) is a strong case study of a successful national FGM healthcare training and curricular programme. Established in 1997, the NZFGMP has worked in collaboration with, and health professionals delivering robust healthcare training and prevention initiatives.

**Methods**

The development of a comprehensive range of pre and postgraduate national FGM curricular, clinical guidelines, web material, resources and recommended best practices. Methods used included FGM research both qualitative and quantitative on the health care experiences of women with FGM. We conducted national FGM training projects on the management and prevention of FGM/C for a wide range of health professionals and medical/nursing universities. We also conducted national FGM health promotion campaigns including consultation, training and community driven FGM programmes. All projects were reviewed using “Results Based Accountability (RBA)” to monitor, evaluate and inform practice.

**Results**

We found improved sexual and reproductive health outcomes of women with FGM (increased satisfaction with maternity services and increased FGM knowledge amongst health professionals). There was a decline in FGM support (from 76% to 43% for type 1 and from 54% to 0% for types 2 and 3) and increased prevention of FGM in New Zealand.

**Conclusion**

The NZFGMP has seen improved sexual and reproductive health outcomes and a decline in support for FGM amongst communities, as a result of the Programme’s consultation, training, resources and community driven initiatives undertaken in close collaboration with communities affected by FGM and health professionals.

### 4 Evidence to inform education, training, and supportive work environments for doctors and midwives involved in the care of women with female genital mutilation: a review of global experience

#### Angela Dawson^1^, Nesrin Varol^2^

##### ^1^The Australian Centre for Public and Population Health Research, Faculty of Health, The University of Technology Sydney, Sydney, Australia; ^2^ Sydney Medical School, University of Sydney, Sydney, Australia

###### **Correspondence:** Angela Dawson (angela.dawson@uts.edu.au)

**Introduction**

There is little available knowledge to inform the design of medical and midwifery education programs or supportive workplace practices in low-, middle-, and high- income countries with respect to caring for pregnant women with female genital mutilation (FGM). We undertook two systematic reviews [1, 2] to examine the experiences and educational needs of doctors and midwives.

**Methods**

Narrative syntheses of peer reviewed primary research literature retrieved through searches of electronic bibliographic databases between 2004 and 2014 were undertaken and guided by the Preferred Reporting Items for Systematic Reviews and Meta-Analyses (PRISMA) framework.

**Results**

A lack of health professional technical knowledge, clinical skills, and limited cultural competency was identified. Studies examining the impact of educational programs are limited and providers in FGM prevalent countries face socio-cultural challenges with respect to the prevention of the practice.

**Conclusion**

There is a need for improved medical and midwifery education and training to build knowledge and skills, and to change attitudes concerning the medicalization of FGM and reinfibulation. Supportive working environments sustained by guidelines and responsive policy and community education are necessary to enable doctors and midwives to improve the care of women with FGM and advocate against the practice.

**References**

1. Dawson A, Turkmani S, Fray S, Nanayakkara S, Varol N, Homer C. Evidence to inform education, training and supportive work environments for midwives involved in the care of women with female genital mutilation: A review of global experience. Midwifery. 2015 Jan 31;31(1):229-38.

2. Dawson A, Homer CS, Turkmani S, Black K, Varol N. A systematic review of doctors’ experiences and needs to support the care of women with female genital mutilation. International Journal of Gynecology & Obstetrics. 2015 Oct 1;131(1):35-40.

### 5 Is research data used in education for health professionals on management of Female Genital Cutting?: results from Sweden

#### Birgitta Essén^1^, Sara Johnsdotter^2^

##### ^1^Uppsala University. Uppsala, Sweden; ^1^Malmö University, Malmö, Sweden

###### **Correspondence:** Birgitta Essén (birgitta.essen@kbh.uu.se)

**Introduction**

Since the 1980s, refugees have come to Sweden from the Horn of Africa, where the majority of women have undergone Female Genital Cutting (FGC). Sweden was the first country in the West to outlaw the practice in 1982, later it became illegal for a Swedish resident to perform FGC abroad. The Swedish government has allocated research funding and has put a lot of effort into prevention and management by means of writing guidelines and organized professional training activities for best practices of persons with FGMC.

**Methods**

We have explored to what extent empirical data from the international research field is used in professional education and policy documents from Swedish health authorities since the 1990s. We performed a systematic review of scientific papers, books, guidelines and grey literature reports from Sweden. We focus on outcomes related to maternity care (maternal, perinatal mortality) and youth health counselling (menstruation disorders, sexuality).

**Results**

The evidence-based knowledge from perinatal and maternal death audits had not been used in an appropriate way, basic medical knowledge on dysmenorrhea was neglected, and qualitative data on sexual health among women with FGC was not acknowledged in the documents.

**Conclusion**

Swedish authorities have worked to improve the health outcomes among women with FGC. However, evidence-based knowledge has been underused, thereby increasing the risk for harm in spite of good intentions.

### 6 Drivers for FGM medicalization among community midwives in River Nile and Northern State, Sudan

#### Paul Bukuluki^1^, Wisal Ahmed^1^, Al Gasseer Naeema H^1^. Dalya eltayeb^2^

##### ^1^World Health Organization, Khartoum, Sudan; ^2^ Federal Ministry of Health (Maternal Child Health Directorate and Human Resource Directorate -CPD), Khartoum, Sudan

###### **Correspondence:** Paul Bukuluki (pbukuluki@gmail.com)

**Introduction**

It is argued that perpetuation of harmful practices, such as female genital mutilation (FGM), may be due to social and economic motivations. At present, in some regions of Sudan, a majority of FGM/Cs are performed by fully trained health professionals, essentially midwives [1]. The reasons (or causes) of this situation need to be understood to ensure appropriate action.

**Methods**

A quality improvement method to optimize Female Genital Mutilation (FGM) In-service Training Package targeting Community Midwives in Northern and River Nile States in Sudan is currently underway with several phases elaborated in a previously published method’s paper [2]. This abstract addresses the formative stage results that explored the socio-cultural, socio-economic drivers, and decision-making processes of community midwives involved in FGM and re-infibulation. We conducted a desk review of existing literature as well as 32 key informant interviews with health workers and community leaders. Eight focus Group Discussions and in-depth interviews were conducted with community midwives.

**Results**

Medicalization is primarily driven by the demand motivated by social norms and the supply motivated by economic gains tending towards commercialization of FGM among the community midwives. It is also driven by limited understanding among community midwives of the depth of Sexual and Reproductive Health consequences of FGM.

**Conclusion**

Programs to reduce medicalization should address both the demand and supply factors. FGM prevention should combine social norm changes and trainings with livelihood options.

**References**

1. In-depth Analysis of MICS and SHHS " Female Genital Mutilation/Cutting (FGM/C) and Child Marriage in Sudan - Are there any changes taking place?" UNICEF 2016

2. Wisal Ahmed et al (2017). Quality Improvement Method to optimize Female Genital Mutilation (FGM): Protocol for an In-service Training Package targeting Community Midwives in Northern and River Nile States in Sudan

### 7 Medicalization of Female Genital Mutilation/Cutting: what do the data reveal?

#### Bettina Shell-Duncan^1^, Caroline Njue^2^, Jacinta Muteshi^2^

##### ^1^University of Washington, Seattle, Washington, USA; ^2^Population Council, Naironi, Kenya

###### **Correspondence:** Bettina Shell-Duncan (bsd@uw.edu)

**Introduction**

Despite international consensus that FGM/C is a violation of human rights, a focus on medicalization remains salient because of concerns that in certain countries FGM/C continues to be performed by healthcare professionals, and may be impeding progress toward abandonment of FGM/C.

**Methods**

We drew on a nationally representative survey data from 25 countries and asked: What are the major patterns and trends in medicalization? What is the association between medicalization and prevalence of or support for FGM/C? What is the association between medicalization and rates of abandonment of FGM/C?

**Results**

Among women between ages 15-49, medicalization is highest in 5 countries: Egypt (38%), Sudan (67%), Guinea (15%), Kenya (15%) and Nigeria (13%). Comparing mothers and daughters, rates of medicalization are rising substantially in all of these countries except Nigeria. Nearly 15 million women in the 25 countries with data on medicalization have been cut by healthcare professionals. Of these, 51% live in Egypt and 34% live in Sudan. Overall, there is no discernible association between rates of medicalization and rates of decline in prevalence of FGM/C and no discernible association between rates of medicalization and support for continuation of FGM/C. Although data are limited, it appears that medicalization is associated with a trend toward less severe forms of cutting (away from infibulation and toward nicking).

**Conclusion**

Medicalized cutting is concentrated in three countries; 93% of women who report having been cut by a health care professional live in Egypt, Sudan and Nigeria. Elsewhere medicalized cutting is rare, or restricted to geographically defined pockets. Medicalized cutting can occur alongside declining prevalence of FGM/C, and hence does not appear to completely counteract abandonment of the practice. The degree to which it potentially slows abandonment or influences the ability of medical care practitioners to participate in anti-FGM/C advocacy is unclear, and requires further research.

### 8 A clinical pathway of sensitization and care for pregnant women with FGM/C in a level II hospital

#### Clotilde Lamy^1^, Pascale Neyrinck^1^, Fabienne Richard^2^, Peter Verduyckt^3^, Sophie Alexander^4^

##### ^1^Iris Sud Hospitals, Brussels, Belgium; ^2^GAMS Belgium, Brussels, Belgium; ^3^ Brussels Health and Social Observatory, Brussels, Belgium; ^4^Université Libre of Brussels, Brussels, Belgium

###### **Correspondence:** Clotilde Lamy (clamy@his-izz.be)

**Introduction**

The HIS maternity unit in Brussels is a level II structure of 2500 annual births serving a largely (approximately 75%) migrant population with a broad component of patients from countries where FGM/C is prevalent.

**Methods**

In this context, pregnancy and childbirth care providers have been trained in identification, classification, obstetrical aspects, management, and prevention of risk of FGM/C in the unborn child. A Train the Trainer course was organized at the Group for the Abolition of Sexual Mutilation (GAMS) and was attended by 4 members of staff, who then conducted training of all health care providers and health visitors of the department.

**Results**

A clinical pathway was established based on three principles: (1) all care providers are competent to identify and classify FMC/C, (2) a large map with incidence per country is posted on the wall of all the consulting rooms, (3) there is a compulsory “FGM/C” box to be checked in the electronic file. The care-giver is responsible for tailored maternal care and prevention of FGM/C in the unborn child.

The Health Information System data were compared to the estimated indirect prevalence using maternal country of origin and provided by civil registration of births. This showed that 72 patients had been identified and cared for within the pathway out of a theoretical estimate of 99.

**Conclusion**

**T**he clinical pathway introduced into maternity leads to a good identification and care of pregnant women with FGM/C by the obstetric team.

### 9 Knowledge of Female Genital Mutilation/Cutting (FGM/C) among nurse-midwives working in high-prevalence counties in Kenya: pre-post KAP study

#### Samuel Kimani^1,2^, Tammary Esho^2,3^, Violet Kimani^1,2^, Christine Kigondu^1,2^,Joseph Karanja^1,2^, Jaldesa Guyo^1,2^

##### ^1^University of Nairobi, Nairobi, Kenya; ^2^Africa Coordinating Centre for Abandonment of Female Genital Mutilation/Cutting, Nairobi, Kenya; ^3^Technical University of Kenya, Nairobi, Kenya

###### **Correspondence:** Samuel Kimani (tkimani@uonbi.ac.ke)

**Introduction**

Female Genital Mutilation/Cutting is implicated in health impacts, women’s rights violation and abuse. Capacity building for Nurse-Midwives as care givers and change agents rather than providers of medicalized FGM/C is critical. We determined the level of knowledge on FGM/C among Nurse/Midwives from FGM/C prevalent counties.

**Methods**

Nurse-Midwives (n = 26) selected from FGM/C prevalent counties took an objective pre/post-quiz administered before and after a 3-day training. The quiz assessed FGM/C key themes namely: definition, classification, perpetuating factors, epidemiology, medicalization/its prevention, health consequences, and Nurse-Midwives’ roles. The themes formed the components covered in the 3-day training. The individual and overall scores for all the questions were computed and compared across the quizzes.

**Results**

The overall mean scores on the quiz were 64.8% before and 96.2% post-training. The scores on specific FGM/C components were; practice types (84.6%), link between cutting and health problems (96.2%), complications (96.2%), cutting communities (61.5%), knowledge of medicalization (43.6%), re-infibulation (46.2%), dissociation from religion (46.2%), and illegality of cutting (46.2%), before training. The performance on FGM/C complications was: physical (69.2%), psychological (69.2%), sexual (57.7%), and social (38.5%), before training. Moreover, the participants awareness of their roles in FGM/C interventions included; counsellor (69.2%), advocate (80.8%), leadership (26.9%), role model (42.3%), and caregiver (34.6%) before training. The scores on all FGM/C themes improved significantly after the 3-day training.

**Conclusion**

Nurse-Midwives exhibited knowledge gaps on FGM/C that may affect their capacity to manage and prevent the practice. This underscores the need to develop and rollout innovative training interventions such as implementing the etool approach on salient issues on FGM/C.

## II. Healthcare and prevention

### 1 FGM: healthcare experience and prevention efforts in Mali: cross sectional study

#### Moustapha Touré^1^, Yacin Gackou Guindo^2^, Dramane Samaké^3^, Ladji Camara^4^, Youssouf Traoré^5^, Alassane Traoré A^6^, Alou Samaké^6^

##### ^1^Mali University Hospital, Bamako, Mali; ^2^National program against female circumcision, Bamako, Mali; ^3^Marie Stoppes International, Bamako, Mali; ^4^Ministry of Environment, Bamako, Mali; ^5^Gabbriel Touré University Hospitals, Bamako, Mali; ^6^Referral Health Center. Commune 6, Bamako, Mali

###### **Correspondence:** Moustapha Touré (mtouregandhi@gmail.com)

**Introduction**

The prevalence of female genital mutilation (FGM) of women aged 15 to 49 years in Mali is very high (91%). The objective of this study was to evaluate the current practice of FGM among children aged 0 to 15 years old.

**Methods**

Cross sectional study of 898 girls between 0 and 15 years old in 3 villages where the Mali Red Cross and NGOs are active. The NGOs work on different health domains including FGM/C. They work on healthcare professionals training and to increase sensitization on the topic among the communities. Participants underwent a clinical examination and their parent/legally responsible person in charge answered a questionnaire.

**Results**

The prevalence of FGM/C was 56%, similar to that of the Demographic and Health Survey, which was 58% for the age group concerned. It was lower in the areas covered by an NGO; 45.5% of children were circumcised before their first birthday. Excision was performed at the parents' home in 56.9% of cases. The two main reasons for performing FGM/C was respected traditions (46.4%) and religion (20%.) The decision-makers for excision were mainly the grandmothers (56.9%) and mothers (31.9%). The practice was performed by medical staff in 2.4% of cases. The main complications were: secondary infibulation/dysuria (54.1%) and vulval cysts (16.2%). The majority (73.6%) of respondents were against a law penalizing the practice; 26.4% in favour.

**Conclusion**

The prevalence of the practice of FGM among children from 0 to 15 is still high in the villages studied. We noted a positive impact of sensitization in areas covered by NGOs, where prevalence was lower than the non-covered areas. Individuals in these communities did not seem to be in favour of a law penalizing the practice. Intensification of awareness campaigns may be useful.

### 2 FGM/C: healthcare experiences and prevention efforts from the U.S. context: mixed methods, community-based participatory research

#### Crista E Johnson-Agbakwu^1,2^ (cejohn11@asu.edu)

##### ^1^Founding Director, Refugee Women’s Health Clinic, Maricopa Integrated Health System, Obstetrics & Gynecology, Phoenix, Arizona, USA; ^2^Assistant Research Professor, Arizona State University, Southwest Interdisciplinary Research Center, Phoenix, Arizona, USA

Since 1970 the United States has experienced a rapid growth of African-born immigrants, with over 1.7 million current residents. The CDC estimates that 513,000 women and girls have undergone or are at risk of FGM/C [1]. FGM/C-affected women possess profound distrust of the health care system, experience stigmatization, face language barriers, and fear interventions; therefore, they often delay or refuse needed care. This may result in adverse reproductive health outcomes [2,3,4,5]. Providers possess widespread knowledge gaps and lack the formal training and cultural knowledge on FGM/C-related care.

A nationally recognized best practice model for improving culturally competent care for FGM/C-affected populations and engaging in Community-Based Participatory Research (CBPR) exists at the Refugee Women’s Health Clinic (RWHC), Maricopa Integrated Health System in Phoenix, Arizona [6]. Cultural Health Navigators, who are employed, bi-cultural, and multilingual staff, facilitate the coordination of culturally competent care, services and support. An infrastructure of community partnership, engagement, and shared community leadership exists through the Refugee Women’s Health Community Advisory Coalition, which is comprised of ethnic community-based organizations, refugee resettlement agencies, as well as public health and academic partners. These entities partner with the RWHC to address FGM/C-associated reproductive health disparities, enhance provider training and clinical documentation and the provision of culturally informed health care that is built on a foundation of established trust, enhanced clinical care, CBPR, and community engagement.

The first ever U.S. “End Violence Against Girls: The Summit on FGM/C” was held in Washington, DC in December 2016, bringing together multiple stakeholders from across governmental agencies, the health, social service, legal and education sectors, as well as FGM/C survivors, community activists, youth, men, and religious leaders [7]. The Healthcare Sector Working Group of the Summit proposed key strategies to respond to FGM/C in the U.S. Multi-pronged efforts are needed that mobilize FGM/C-affected communities and providers in addressing the social determinants of health, ameliorating structural barriers to care, engaging men as partners, enhancing sustained provider education, and supporting the dissemination of inter-professional clinical practice guidelines. Future research must utilize validated instruments, provide ethno-cultural specificity, incorporate WHO FGM/C typology, design quality improvement metrics, and encourage multi-center research partnerships.

**References**

1. Goldberg H, Stupp P, Okoroh E, Besera G, Goodman D, Danel I. Female Genital Mutilation/Cutting in the United States: Updated Estimates of Women and Girls at Risk, 2012. Public Health Reports. 2016; 131:1-8.

2. Johnson CE, Ali SA, Shipp MP-L. Building Community-based Participatory Research Partnerships with a Somali Refugee Community. Amer J Prev Med 2009; 37(6S1): S230-S236.

3. Johnson-Agbakwu CE, Helm T, Killawi A, Padela A. Perceptions of Obstetrical Interventions and Female Genital Cutting: Insights of Men in a Somali Refugee Community. *Ethnicity & Health,* 2014; 19(4): 440-457. http://dx.doi.org/10.1080/13557858.2013.828829

4. Lazar JN, Johnson-Agbakwu CE, Davis OI, Shipp MP-L. Challenges in Obstetrical Care of Somali Women: A Qualitative Study of Provider Perspectives. *Obstetrics and Gynecology International,* 2013; http://dx.doi.org/10.1155/2013/149640

5. Fawcett L. (2014) Somali Refugee Women and Their U.S. Healthcare Providers: Knowledge, Perceptions and Experiences of Childbearing. Doctoral Dissertation, Arizona State University.

6. Agency for Healthcare Research and Quality. Community-Driven Clinic for Refugee Women Enhances Access to Comprehensive, Culturally Sensitive Care Across the Reproductive Life Span | AHRQ Innovations Exchange. *AHRQ*. 2014. Available at: https://innovations.ahrq.gov/profiles/community-driven-clinic-refugee-women-enhances-access-comprehensive-culturally-sensitive Accessed April 19, 2017.

7. U.S. End Violence Against Girls: Summit on FGM/C. Washington, DC, 2016. https://www.usip.org/sites/default/files/files/Agenda-FGMC.pdf Accessed April 19, 2017.

### **3 Clitoral reconstruction (CR) after female genital cutting (FGC). Women’s motives, expectations and experiences**: **qualitative study**

#### Malin Jordal (malin.jordal@gender.uu.se)

##### Center for Gender Research, Uppsala, Sweden

**Introduction**

Clitoral reconstruction (CR) has recently been introduced in Sweden.

**Methods**

Qualitative study aimed at exploring motives, expectations, and experiences in relation to surgery.

**Results**

Fifteen women requesting surgery were recruited at Karolinska University Hospital. Preliminary analysis of 15 pre-operative and 4 post-operative semi-structured individual interviews revealed that the women feel FGC has deprived them of something important for their sexual capacity, resulting in grief and feelings of inferiority. They wanted to reclaim this body part, physically and symbolically, although aware that surgery might not ‘fix everything’. They hoped their genitalia after surgery would resemble uncut genitalia so that they could look and feel more ‘normal’. They also hoped to regain clitoral sensation and improve sexual capacity. This, they reported, could make them more ‘equal’ with uncut women. One year post-operatively, the women were satisfied with surgery on at least some levels, despite strong immediate post-operative pain. Improved sexual pleasure and a newfound ability to reach orgasm, was experienced, but some women reported no difference in sexual pleasure and capacity. The women were satisfied with the visual aspects and happy to have gone through surgery.

**Conclusion**

The complexities involved in CR, including social, psychological and emotional aspects, should be taken into consideration in future studies.

### 4 FGM/C as a health concern - lay people’s views on the bodily practice in Burkina Faso: an ethnographic study

#### Elena Jirovsky (elena.jirovsky@meduniwien.ac.at)

##### Medical University of Vienna, Center for Public Health, Department of General Practice and Family Medicine, Vienna, Austria

**Introduction**

In Burkina Faso, there is a legal ban of FGM/C. Nationwide anti-FGM/C campaigns tackling related health and social issues aim at societal change. Different media, such as TV, radio, films, theatre, forum discussions, and music videos, are used to spread information. This study was conducted to identify current social and cultural perceptions, validations, and norms pertaining to this traditional, yet contested, cultural practice.

**Methods**

Ethnographic, qualitative fieldwork has been conducted in Bobo-Dioulasso, Burkina Faso. A narrative approach was used to elicit how people subjectively explain and make sense of FGM/C. Participant observation with informal interviews, as well as expert interviews with stakeholders of the campaigns were conducted. Furthermore, semi-structured interviews were made with 55 female and male city dwellers of various religions and ethnic origins between the ages of 18 and 55+ years.

**Results**

Amongst others, health, and disease, physical inadequacy, or physical problems are part of the public discourse on FGM/C. People debate and contest the reality of health consequences of the practice in the framework of related gender notions, e.g. the view of the circumcised body, which was overall considered to be healthy and imperative for the wellbeing of the individual woman, her family, and society at large, is changing. It is now also viewed as an abnormal, unhealthy, incomplete and perilous matter for both the cut individual and her socio-environment. (Table 1)

**Table 1 (abstract 4):** Examples of quotes. All names used in this table are pseudonyms

Speaker	**Interview verbatim transcripts**
Female speaker “Awa” ^1^, 23-years-old, market vendor, married, 2 children	*“All women, at a certain moment, are up to make love. If this need comes, it does not manifest equally for every woman. It is said that if a woman is circumcised and feels this mood for sex, she can sustain it. But those who are not circumcised, if they feel this need, their vagina tempts them. She cannot stay calm if she does not make love.”*
Male speaker “Haroun” 39-years-old, unemployed photographer, married, 4 children	*“It was said, ‘A woman who is not circumcised, she always wants to make love. The moment she is in bed with her husband, it is tough. The man has to make an effort. […] Effectively, later others said, ‘Cut away there and you have to stimulate her tirelessly, and it does not mean anything for her. In bed, they are losers.’ Others even say that it even hurts them. That is, it cannot heal just like that.”*
Male speaker “Moises”, 27-years-old, unemployed, unmarried, 1 child	*“If they were cut, it could help them to better control themselves. I don’t like [the uncut girls’] behavior. If we could instruct [the ones performing excision] and give them rules for circumcising the girls, I would view this as a good thing. Because like this, they could catch a disease… […] It is not sure whether thse girls can stay faithful to their husbands”*
Female speaker “Fatoumata”, 18-years-old, high-school student graduation class, unmarried, no children	*“What they have to establish, in my view, is to circumcise the young in the hospital, and to avoid traditional female circumcision. It is not good. It is risky. Especially the rusty knives and all that… it elicits illnesses in the genital apparatus of the woman. The boys, there are no consequences. But the girls have consequences due to the cutting. They have to establish one law for all the young, boys and girls alike, so that they all are circumcised in the hospital..”*
Male speaker “Boureima”, 44-years-old, tailor, married, 3 children	*“[…]in the past, it was possible to lose blood [during excision]. Going to the hospital, you would find blood to give fast. But nowadays, when you lose blood, to have good blood to replace it in the body, this would not be easy. I have seen it myself, but not in connection to excision. There is a woman, who […] got blood, but found that the person had hepatitis. She died[…]Nowadays one should not take that risk. When you do the cutting, if the girl loses a lot of blood, in the hospital, this will not be easy.”* *[…]* *“In former days, there wasn’t AIDS and then the diseases like that. There weren’t any. And then, the people, everything is fragile nowadays. The food is bad. […] In the past, those who practiced the cutting, they […], they did it in a way that was limited. Now, because of the money, those who don’t know, they entered into this trade. That brings problems.”*
	*“I prefer those who are not excised. If by chance, I find a woman who is cut, I do not mind either. This is faith. But the preference, I prefer the girls who are not cut, because they are as God created them.”*
Male speaker “Le Lion”, 22-years-old, market vendor, unmarried, 1 child	*I consider excision as a crime. It is the mangling of bodies, which God has* *created. It is an enormous pain, which influences the life of the small girls.* *[..] They are mangled, they are cut, and this hurts me. I do not like it. I* *fight against it.*

**Conclusion**

Participants seemed to view FGM/C as a health topic and it was discussed as a health concern. It is possible that this was due to the strong emphasis on health-related issues in the campaigns. As shown in the narratives, both proponents and opponents internalised the health arguments of the campaigns against FGM/C. Both deemed it as a negative that FGM/C might result in poor health outcomes.

### 5 Improving FGM/C healthcare and clinical guidelines through community engagement and leadership: a review

#### Samantha Wu^1^, Kevin Fitzgerald^1^, Ranit Mishori^2^, Rebecca Reingold^3^

##### ^1^Pellegrino Center for Clinical Bioethics. Georgetown University Medical Center. Washington DC, USA

^2^Department of Family Medicine, Georgetown University School of Medicine, MedStar Health – Family Medicine at Spring Valley, Washington DC, USA

^3^O’Neill Institute for National and Global Health Law, Georgetown University Law Center.

Washington DC, USA

###### **Correspondence:** Samantha Wu (sw462@georgetown.edu)

**Introduction**

There is a growing recognition of the need to engage communities and individuals in the effort to improve health outcomes, especially among those at risk of and survivors of female genital mutilation/cutting (FGM/C). As part of a larger project aimed at developing more ethical, culturally-competent clinical guidelines for FGM/C, we seek to develop a set of key elements for effective engagement of women and girls at risk of FGM/C, based on a review of current and ongoing efforts across a variety of settings.

**Methods**

We reviewed a total of 22 organizations working with those at risk and survivors of FGM/C in the United States, the United Kingdom, Uganda, The Gambia, Guinea, and Kenya. Recognizing the importance of interdisciplinary approaches to improving health, we looked at organizations in healthcare (5), community-based education and training (7), advocacy and support (9), and legal services (1). Organizational goals, programming, and resources were included in the review.

**Results**

Key elements identified could be categorized into three areas: promoting communication and dialogue, tailoring healthcare to community needs, and forming sustainable partnerships. Elements of effective communication and dialogue included: a) creating spaces and platforms where goals, values, and needs of community members can be made explicit, and b) recognizing the unique context of community members, so as to avoid judgment, labeling or blame. Those related to healthcare included: a) tailoring training for health professionals to facilitate understanding of the type, history, consequences and significance of FGM/C in specific communities, b) advocating for the development of clinical and professional guidelines that reflect community needs & women’s empowerment, c) developing clearly defined, system-wide referral procedures, and d) implementing continual monitoring and evaluation procedures. The final set of elements relate to forming sustainable partnerships: a) integrating community assets and characteristics (strengths, history, and limitations), b) recognizing, prioritizing, and promoting diversity in leadership, c) engaging and empowering communities to take responsibility for change; supporting survivor-led and community-based efforts, d) implementing concrete feedback and evaluation procedures, and e) requiring a research component to measure change and validate progress.

**Conclusion**

Current efforts to address FGM/C are multidisciplinary and diverse, but have key elements in common. These key elements may be used to engage communities in the development and implementation of organizational programming, public health interventions, and clinical guidelines that work with those survivors of and those at risk of FGM/C.

### 6 The role of midwives at the Edna Adan University Hospital to prevent Female Genital Mutilation: comparative prevalence study, 2002 to 2006 and 2007 to 2015

#### Edna Adan Ismail (ednahospital@yahoo.com)

##### Edna Adan Hospital, Hargeisa, Somaliland, Somalia

Fifteen years ago, the Edna Adan Hospital was opened to provide services to the public as well as a platform to assess the real situation about the prevalence and types of FGM in the community the hospital served. The results that emerged from the data collected from the first 4000 women attending our prenatal care between 2002 to 2006 showed that after forty years of campaigning, FGM was very much entrenched in the culture of the people in spite of efforts that had been deployed in the past.

Having pioneered the fight against FGM in 1976, the disappointing result prompted us to deploy new and additional strategies to protect girls from female genital mutilation.

We realized that efforts in the past were being made by only by a few individuals who were willing to take risks to speak about FGM in public and what was now needed was a way to have as many people as possible to be given the necessary information about FGM who could then speak about it with confidence.

This made me take the decision to enforce the regulation in my hospital that all nurses, midwives and doctors we train must learn more about the harmful effects of FGM, and also agree to speak publicly about it if they wished to remain in our courses.

The result of a second survey carried out on the next 6,000 women seeking prenatal care at the Edna Adan Teaching Hospital revealed that the incidence of type 3 FGM had decreased from 99% of the women attending prenatal care between 2002 to 2006 to 76% type 3 affecting the women seeking prenatal care from 2007 to 2015.

Increasing care givers’ education on harmful effects of FGM/C and engaging them in adocacy was associated with a 30% decrease in Type 3 FGM/C among women attending the Edna Adan Teaching Hospital.

## III. Knowledge, Evidence and Consensus Gaps

### 1 Implementation of the guidelines on management of complications of FGM, research gaps and research implications: a commentary

#### Lale Say (sayl@who.int)

##### Department of Reproductive Health and Research, World Health Organization, Geneva, Switzerland

WHO plays an important role in strengthening a health sector response to FGM, including the development of evidence-based guidance and practical tools for healthcare providers; supporting countries to implement guidance and generating evidence to inform policy and programs related to the health sector. The WHO Guidelines on the Management of Health Complications from FGM - published in May, 2016 - provide up-to-date, evidence-informed recommendations on the management of health complications from FGM^1^. The guidelines were developed using standard WHO guideline development operating procedures, which involve a rigorous, step-by-step process with multiple levels of review by internal and external committees and experts. The process resulted in the formulation of 5 recommendations and 8 best practice statements grouped in four main areas: (1) deinfibulation to prevent and treat obstetric complications, and urological conditions; (2) mental health; (3) female sexual health; and (4) information and education interventions for both healthcare providers and women living with FGM. Using these guidelines as a foundation, WHO is currently developing training materials for healthcare providers, which will be developed and tested as part of an implementation research process to prevent the medicalization of FGM and improve the care for women and girls living with FGM [1].

WHO. WHO Guidelines on the Management of Health Complications from Female Genital Mutilation. Geneva: World Health Organization 2016.

### 2 A pilot study on pelvic floor symptoms in women living with female genital mutilation/cutting: preliminary results

#### Marion Uebelhart^1^, Michel Boulvain^2^, Patrick Dallenbäch^2^, Olivier Irion^2^, Patrick Petignat^2^, Jasmine Abdulcadir^1,2^

##### ^1^Faculty of Medicine, University of Geneva, Geneva, Switzerland; ^2^Department of Obstetrics and Gynecology, Geneva University Hospitals, Geneva, Switzerland

###### **Correspondence:** Jasmine Abdulcadir (jasmine.abdulcadir@hcue.ch)

**Introduction**

Female Genital Mutilation/Cutting (FGM/C) can cause both psychosexual and physical consequences including pelvic floor disorders. FGM/C and pelvic floor disorders are both under diagnosed and undertreated conditions that can significantly impact women’s life quality. The aim of the study is to determine the prevalence of pelvic floor symptoms and disorders among women with FGM/C and test available validated questionnaires.

**Methods**

Cross sectional study started in April 2016, at the Department of Gynecology and Obstetrics of the Geneva University Hospitals on 121 women with different types of FGM/C. Six validated questionnaire scores (PFDI-20, PFIQ-7, PISQ-IR, FGSIS, FISI, and Wexner constipation questionnaire) and sociodemographic information were collected. The questionnaires are administered in French or in English, when needed with a certified and accepted female interpreter. The scores of the questionnaires validation studies on women without FGM/C with or without pelvic floor symptoms were used as reference.

**Results**

Data on 60 women are presented as preliminary results. Fourteen (23%) have FGM/C type 3. The remaining women have FGM/C type 1, 2 or defibulated type 3. Forty five percent of women referred other past traumatic sexual, psychological or physical events different than FGM/C or forced marriage. Women with FGM/C reported questionnaires’ scores indicating a negative impact on the quality of life due to pelvic floor symptoms (PFDI-20 and PFIQ-7) and a lower satisfaction of the genital self-image (FGSIS).

**Conclusion**

Preliminary results indicate that women with FGM/C report scores similar to those of women without FGM/C but who experienced pelvic floor symptoms and disorders.

### 3 The impact of the law in the prevention of FGM: legal analysis

#### Patrizia Farina^1^, Els Leye^2^, Livia Ortensi^1^, Claudia Pecorella^3^

##### ^1^Department of Sociology and Social researchUniversity of Milan Bicocca, Milan, Italy; ^2^International Centre for Reproductive Health, Ghent University, Ghent, Belgium; ^3^Department of Law, University of Milan Bicocca, Milan, Italy

###### **Correspondence:** Patrizia Farina (patrizia.farina@unimib.it)

**Introduction**

Among policies developed to abandon FGM, an important role has been attributed to legal instruments. Within the demand for a comprehensive approach to prevent and protect against FGM, many European Member States have developed specific criminal laws dealing with FGM and provided civil measures for protecting minors. However, the implementation of these laws seems to be weak and adequate and complete information concerning the actual effect of the law in the prevention of FGM is lacking. Using the 2016 Italian Sampling Survey, developed under the EU Daphne Project (“methodology for estimating FGM prevalence in Belgium and Italy”). We assessed: a) how much knowledge of laws against FGM existed among migrants and b) if and how being aware of these laws influenced abandoning FGM.

**Methods**

In the Survey women were asked about their knowledge of the law; moreover, if the criminalization of FGM was included among the possible reasons reported by women who declared their intention not to cut their daughters. The survey ended in December 2016.

**Results**

The majority of women agreed on state intervention to prevent cutting of girls. The pattern of the agreement is similar to Italy and country of birth. Agreement on awareness of the law as a prevention tool was lower among women from Burkina Faso, Egypt, and Nigeria, the communities with the highest FGM prevalence in Italy. As per the knowledge of the law, migrant women know much more about laws in their country of origin than about laws in Italy. Lack of knowledge was higher among Nigerian, Egyptian, and Burkinabe women.

**Conclusion**

Our results suggest that knowing about anti-FGM/C laws could contribute to the abandonment of cutting. However, knowledge of laws was less relevant to women from high prevalence FGM/C communities.

### 4 Persistent norms and tipping points: Female Genital Cutting in Burkina Faso: theory testing

#### Lindsey Novak (novak352@umn.edu)

##### University of Minnesota, Minneapolis, Minnesota, USA

**Introduction**

Female Genital Cutting (FGC) has persisted for generations because deviating from the norm can be costly. The prevailing theory in the study of FGC is that it is a social coordination norm—that is, households will abandon FGC if and only if a sufficient proportion of households within the community agree to abandon the practice. Under this theory, if a sufficient number of community members agree to abandon FGC, a tipping point is reached and FGC could be eliminated. However, recent empirical evidence rejects that theory.

**Methods**

I contributed to this important debate by generating a new theory of why FGC persists, and I tested that theory using a dataset of 7,500 women born between 1949 and 1995 in Burkina Faso.

**Results**

Households within a community have heterogeneous preferences for FGC, such that each household may require a different proportion of community members to abandon FGC before they also reject FGC. This heterogeneity makes the existence of a tipping point uncertain, and stable interior equilibria in FGC rates are possible.

**Conclusion**

My findings suggest that individuals and households are in fact able to deviate from an entrenched, gender-biased social norm and that policies to reduce the prevalence of FGC perhaps should prioritize targeting individual and household, rather than village-level, preferences.

### 5 What is the evidence on safety and efficacy of clitoral reconstruction after FGM/C?: systematic review

#### Jasmine Abdulcadir (jasmine.abdulcadir@hcuge.ch)

##### Faculty of Medicine, University of Geneva, Department of Obstetrics and Gynecology, Geneva University Hospitals, Geneva, Switzerland.

**Introduction**

Clitoral reconstruction or transposition after female genital mutilation/cutting (FGM/C) is a procedure that consists of a resection of the periclitoral fibrous tissue and re-exposition of a healthy clitoral neo-glans. This surgery aims to improve pain symptoms, sexual function, body image, and female identity. However, some recent guidelines do not recommend the procedure due to a lack of conclusive evidence.

**Methods**

A systematic review was performed in 2014 (PubMed and Cochrane) including any design/language study reporting on safety and clinical outcomes of clitoral reconstruction after FGM/C.

**Results**

In addition to the four studies included in 2014, 9 papers were identified. They are fair to poor in quality. They report on immediate and long-term complications and outcomes (clitoral appearance, dyspareunia, clitoral pain, and function) mostly via non-standardized scales, on small samples and with no control groups.

**Conclusion**

Additional multicentre and adequately powered research is needed on this procedure. Futures studies should also focus on context and conditions that may influence the request and acceptability of clitoral reconstruction and on alternatives to non-surgical management (sexual counselling and psychosexual therapy).

### 6 How to evaluate sexual dysfunction in female genital mutilation: a proposed model based on a review of literature and clinical experience

#### Béatrice Cuzin^1^, Florence Brunel Delmas^1^, Albertine Papingui^2^

##### ^1^GH E Herriot, Lyon, France; ^2^GAMS, Lyon, France

###### **Correspondence:** Béatrice Cuzin (beatrice.cuzin@chu-lyon.fr)

**Introduction**

Sexual dysfunction in FGM presents is complex and can include hypoactive desire, decreased arousability and anorgasmia. FGM can also cause chronic urogenital pain and perineal scarring. Clitoral reconstructive surgery can improve some sexual dysfunction, but psychosexual therapies also have an important role to play. However, there is a need for standard evaluation for relevant management.

**Methods**

The available literature on sexual dysfunction after FGM was identified by searching PubMed and Cochrane databases from January 1990 to Dec 31, 2016. Search terms related to FGM and sexual function were used in various combinations.

**Results**

A total of 9 studies evaluated sexual function after FGM with the validated Female Sexual Function Index (FSFI). Seven were cross sectional studies, and two were reports of sexual function after clitoral reconstructive surgery. There were no studies evaluating the impact of psychosexual therapies in FGM. One study used the validated Female Sexual Distress Scale (FSDS). Other studied parameters included depression, body image, and clitoral sensations.

**Conclusion**

There is a need for prospective studies using validated questionnaires to assess the integrative approach of sexual function after FGM. For now, available qualitative data and validated tools used in Sexual Medicine could help in assessing sexual function after FGM.

### 7 Do ‘female circumcision’ and ‘genital cosmetic surgery’ have the same roots? A review of the historical evidence on female genital surgeries in Western Europe

#### Dina Bader (dina.bader@unine.ch)

##### Institute of Social Sciences, University of Lausanne, Lausanne, Switzerland; Swiss Forum for Migration and Population Studies, University of Neuchâtel, Neuchâtel, Switzerland

**Introduction**

In dominant Western public discourses, ‘female circumcision’ and ‘genital cosmetic surgery’ (FGCS) are considered very different types of bodily practices. However, only a few studies have tried to assess the validity of this prevailing conventional wisdom. This research project examined one of the most sensitive questions in the debate over FGCS: Is FGCS identified as, or even conflated with, ‘female circumcision’.

**Methods**

Based on a review of both the literature and historical archives, this paper traces the spatiotemporal evolution of FGCS and examines the reasons given for the practice.

**Results**

The review shows that FGCS is not a new phenomenon but is part of a continuity of bodily practices that have their roots in the classical period. Furthermore, there are sociohistorical connections between ‘female circumcision’ and FGCS and clear references to the former by European physicians performing several types of female genital modification throughout the centuries well into the beginning of the 20^th^ century. In fact, the review reveals that ‘female circumcision’ and FGCS have had and still have a similar capacity to modify female genitalia according to social norms of aesthetics and sexuality.

**Conclusion**

The assertion of a radical distinction between ‘female circumcision’ and FGCS is not sustainable based on the sociohistorical evidence.

### 8 Factors associated with the support of pricking (female genital cutting type IV) among Somali immigrants – a cross-sectional study in Sweden

#### Anna Wahlberg^1^, Sara Johnsdotter^2^, Katarina Ekholm Selling^1^, Carina Källestål^1^, Birgitta Essén^1^

##### ^1^Uppsala University. Uppsala, Sweden; ^2^ Malmö University. Malmö, Sweden

###### **Correspondence:** Anna Wahlberg (anna.wahlberg@kbh.uu.se)

**Introduction**

Pricking, classified as female genital cutting (FGC) type IV by the World Health Organization, is an under-researched practice that appears to be increasing among diaspora communities. Our aim was to explore factors associated with being supportive of pricking among Somalis in Sweden.

**Methods**

In a cross-sectional design, attitudes and knowledge regarding FGC, and measures of socioeconomic status, acculturation, and social capital, were assessed by a 49-item questionnaire in four municipalities in Sweden. Data were collected in 2015 from 648 Somali men and women, ≥ 18 years old, of which 113 supported the continuation of pricking. Logistic regression was used for the analysis.

**Results**

Those more likely to support the continuation of pricking were older, originally from rural areas, and newly arrived in Sweden. Further, those who reported that they thought pricking was: acceptable, according to their religion (aOR: 10.59, 95% CI: 5.44-20.62); not a violation of children’s rights (aOR: 2.86, 95% CI: 1.46–5.61); and did not cause long-term health complications (aOR: 5.52, 95% CI: 2.25–13.52) had higher odds of supporting pricking.

**Conclusion**

Values known to be associated with FGC in general are also related to pricking. Hence, there seems to be a change in what types of FGC are supported rather than in their perceived values.

## IV. E-posters

### Healthcare professionals: training and curricula. Medicalization

#### 1 Dissemination of the WHO guidelines on the management of health complications from Female Genital Mutilation: a commentary

##### Abdalla Hisham Hussein Imam Ibraheim (hhimam@yahoo.com)

###### Faculty of Medicine, Minia University, Minia, Egypt

In May 2016, WHO developed very comprehensive guidelines on the management of health complications of female genital mutilation. This effort needs to be disseminated worldwide to help survivors of FGM and to fight against this severe type of violence against women. It is recommended that we create a task force to work on dissemination of the WHO guidelines. This task force could work on different fronts: First, the task force could communicate with medical institutions worldwide to encourage them to include these guidelines as part of their curricula, Second, the task force could communicate with scientific societies concerned with women’s health to encourage their members to make use of the guidelines. Third, the task force could communicate with educational bodies worldwide to develop an educational module from the WHO guidelines on the management of health complications from FGM and implement this in an in-class and on-line courses. We also need to fight the idea of Medicalization of FGM/C and last, we need to work on conducting the research that is needed to fill the gaps identified during the preparation of the guidelines.

#### 2 Female Genital Mutilation in Sudan - Are obstetricians and gynecologists more ready than pediatricians? Pre-post intervention study, preliminary analysis

##### Nasr A.M. Elawad^1^, Wisal Ahmed^2^, Al Gasseer Naeema H^2^. Elamin Maison^2^, Hiba Hussein^2^, Altayyeb Mohammed Albagir^2^, Paul Bukuluki^2^, Mohamed Tawfig Albirair^3^

###### ^1^Sudan Obstetric and Gynecological Society, Khartoum. Sudan; ^2^World Health Organization Khartoum, Khartoum, Sudan; ^3^Public Health Institute, Khartoum, Sudan

####### **Correspondence:** Nasr A.M. Elawad (nasr.abdalla@gmail.com)

**Introduction**

FGM is an ancient practice in Sudan, of unclear origin and commonly practiced in many Sudanese societies for different reasons. In Sudan, 86.6% of females (15-49 years) have undergone FGM mostly in its most severe form, WHO type III (77%). Secondary FGM defined as re-circumcision in last 12 months is highest among women between 15-19 years (31.2%) compared to 20 - 39 years (23 -24%). Obstetricians and gynaecologists can play an important role at both community and individual level in the prevention and care of FGM/C and against this practice (primary and secondary FGM) and its medicalization. To function effectively clinicans need to acquire knowledge and skills on FGM epidemiology, social norm concepts, and FGM health complications’ management.

**Methods**

Sudanese Obstetricians & Gynecologists (OBGYNs), who attended their annual scientific conference in February 2017, participated in a KAP assessment on various aspects of FGM. Before this event, pediatricians participated in the same assessment. To collect the required data a questionnaire was developed and reviewed by experts. Four senior medical students were nominated by the conference organizing committee and well oriented to the objectives of the survey, the content of the questionnaire, and how to distribute and compile the filled questionnaire. Daily, the organizing committee made an announcement encouraging meeting attendees to complete the questionnaires. One hundred and fifty three OBGYNs completed the questionnaire. The completed questionnaires were reviewed and the data was analyzed with analysis SPSS and followed by interpretation.

**Results**

Preliminary findings showed a higher level of knowledge on FGM and its complications management among the OBGYNs compared to Pediatricians, but similar findings on social norm change. Only 4.5% of the respondents reported the correct FGM prevalence rate in Sudan, 28.8% identified the WHO typology of female circumcision, and 37.2% listed three FGM health complications. Nearly 8% stated that health professionals should perform female circumcision, while 78.8% called for elimination of female circumcision. In terms of FGM practice, 51.3% saw health complications in their clinical practice; 20.5% correctly managed FGM health complications.

**Conclusion**

Obstetricians had more knowledge about FGM and its complications compared to Pediatricians, perhaps because of their greater clinical exposure. Based on the study results, there appears to be a need to incorporate FGM in all the pre-and in-service trainings for doctors, nurses and midwives. This approach can ensure that the concerned health professionals have the minimum requirements to play their key role in combating FGM and to help those who have been cut by appropriately managing their immediate and late health complications. There was only a small number of obstetricians enrolled in this study, so it may not be representative of all Sudanse OBGYNs. In order to have a more representative sample; we propose to conduct a study with a larger sample.

#### 3 Female Genital Mutilation in Sudan: are pediatricians ready to fight this practice? A preliminary analysis of a pre-post intervention study

##### Sarah A. Salam Salih^1^, Wisal Ahmed^2^, Al Gasseer Naeema H^2^,Elamin Maison^2^, Hiba Hussein^2^, Altayyeb Mohammed Albagir^2^, Mohamed Tawfig Albirair^3^, Paul Bukuluki^2^

###### ^1^Sudan Association of Pediatricians, Khartoum, Sudan; ^2^ World Health Organization, Khartoum, Sudan; ^3^ Public Health Institute, Khartoum, Sudan

####### **Correspondence:** Sarah A. Salam Salih (sasalam@hotmail.com)

**Introduction**

Female Genital Mutilation/Cutting prevalence is high (86.6%) in Sudan, mostly carried out in girls between 5-9 years old. Pediatricians can play an important role at both community and individual level in prevention and care of FGM/C and against the practice and its medicalization. We aimed to assess the knowledge, attitude and practices (KAP) of pediatricians on medicalization, prevention, complications management, and social norm change.

**Methods**

Sudanese paediatricians attending their annual conference participated in a KAP assessment on medicalization, prevention and complications management, and social norm change. An intervention that comprised of a presentation package in these assessment areas was provided during the conference session, the intervention included the paediatricians signing a declaration against medicalization and a call for stricter regulatory mechanisms. Data was collected using a semi-structured, self-administered questionnaire with questions on knowledge, attitudes and practices in relation to FGM/C. The effect of this intervention will be assessed in future annual conferences.

**Results**

Analysis of 154 questionnaires (with most of the questions answered >85%) showed that mean age of respondents was 37 years, 52% were females, 96.1% were Muslims, 47.3% were specialists (47.4%) or specialists-in-training (32.5%). A few (9.7%) reported never having received any type of FGM training. Only 2% of pediatricians showed correct knowledge of the 4 types of FGM in Sudan; 60% were able to list 3 WHO FGM complications. Knowledge of correct management of WHO complications was much lower (11%), although 51% managed FGM complications in their practice. The attitudes of pediatricians towards FGM showed that 97% of them would not encourage it, 67.7% thought it needed to be criminalized, and 63% considered the practice a violation of women’s and girls’ rights. Sixteen percent of respondents believed there are religious benefits to it, and 16% said they would have their daughters cut. Attitudes of the general population in other surveys showed similar results with 67% of urban and 45.5% of rural women favoring FGMC abandonment. Logistic regression analysis showed that specialists and specialists-in-training had more knowledge on FGM typology compared to Junior doctors (OR = 2.2e-09; P-value = 0.00). No association was found between previous FGM training and FGM knowledge. Specialists or in-specialist trainees tended to identify FGM as violation of rights compared to junior staff (OR 3.48; P-value 0.03) and (OR 3.16; P-value 0.06) respectively. Results also showed that older age was associated with an increased likelihood of having seen FGM complications and having correctly managed them per WHO guidelines.

**Conclusion**

This study identified knowledge, attitudes and skills gaps and the need to integrate FGM training content within medical and specialization curricula. Concepts of social norm change also needs to be strengthened among pediatricians.

#### 4 Continuing professional education on Female Genital Mutilation for obstetricians, gynecologists, and midwives in Australia: educational program development

##### Angela Dawson^1^, Nesrin Varol^2^

###### ^1^The Australian Centre for Public and Population Health Research, Faculty of Health., University of Technology Sydney, Sydney, Australia; ^2^Sydney Medical School, University of Sydney, Sydney, Australia

####### **Correspondence:** Angela Dawson (angela.dawson@uts.edu.au)

**Introduction**

Australia has experienced a growth in the number of women and girls arriving from countries where Female Genital Mutilation (FGM) is prevalent. Obstetricians, gynaecologists, and midwives are therefore caring for increasing numbers of affected women, highlighting the need for additional education and training. We developed the first nationally accredited continuing professional development program for the Royal Australian and New Zealand College of Obstetricians and Gynaecologists.

**Methods**

An expert panel of medical and midwifery clinicians, social scientists, and educators formed a consortium to develop and trial four on-line training modules.

**Results**

The online modules introduce health professionals to the issue of FGM in Australia, outline the sexual and reproductive health consequences of FGM and address the care and clinical support that women require. Information to support education and advocacy is also provided. An evaluation found the modules to be relevant and applicable to clinical practice. Fifty-five of the 61 specialists who provided feedback since mid-2016 have rated the modules as “very good” or “excellent”. Qualitative comments noted the importance of graphics and videos in preparing to manage the next patient with FGM, and also noted the usefulness of the modules as part of an orientation for volunteer work in high prevalence FGM countries. In one State this training has been supported by clinical guidelines and a healthcare professional counselling aid.

**Conclusion**

A national approach to training and education for Australian health professionals was an important part of preparing clinicians to deliver optimal care to pregnant women with FGM. However, supportive workplace environments also are needed to ensure learning can be applied in practice.

## V. Healthcare and prevention

### 1 “The ‘heat’ goes away”: sexual disorders among married women with Female Genital Mutilation/Cutting in Kenya: a mixed methods study

#### Tammary Esho^1,2*^ Samuel Kimani^1,3^, Violet Kimani^1,4^, Samuel Muniu^1^, Christine Kigondu^1,5^, Isaac Nyamongo^1,6^, Jaldesa Guyo^1,7^, Patrick Ndavi^1,7^

##### ^1^African Coordinating Centre for Abandonment of FGM/C, University of Nairobi, Kenyatta National Hospital, P.O Box 19676-00202, Nairobi, Kenya; ^2^ Department of Community and Public Health, Technical University of Kenya, P.O Box 52426, Nairobi, Kenya; ^3^ School of Nursing sciences, Kenyatta National Hospital, P.O Box 19676-00202, Nairobi, Kenya; ^4^School of Public Health, Kenyatta National Hospital, P.O Box 19676-00202, Nairobi, Kenya; ^5^Department of Clinical Chemistry, University of Nairobi, Kenyatta National Hospital, P.O Box 19676-00202, Nairobi, Kenya; ^6^Institute of Anthropology, gender and African studies, University of Nairobi, Nairobi, Kenya; ^7^ Department of Obstetrics and Gynaecology, University of Nairobi, Kenyatta National Hospital, P.O Box 19676-00202, Nairobi, Kenya

###### **Correspondence:** Tammary Esho (esho.tammary@tukenya.ac.ke)

**Introduction**

There is paucity of research investigating sexual experiences among married women with Female Genital Mutilation/Cutting (FGM/C). This study investigated the sexual experiences among married women in Kenya.

**Methods**

The study used a mixed methodology. A total of 318 married women enrolled were categorized into three clusters; those cut before marriage, those cut after marriage, and those who married uncut. Data was collected using a psychometric instrument (FSFI), entered and analysed using SPSS Version 22. Focus group discussions, interviews and case narratives were also conducted. Data was transcribed verbatim, thematized, analyzed and interpreted.

**Results**

The reported overall sexual functioning was significantly (p = 0.019) different across the three groups. Women cut after marriage (mean = 22.81 ± 4.87) scored significantly lower (p = 0.056) than women who were uncut (mean = 25.35 ± 3.56). However, in comparison to those cut before marriage, there was no significant difference (mean = 23.99 ± 6.63). Among the sexual functioning domains, lubrication (p = 0.008), orgasm (p = 0.019) and satisfaction (p = 0.042) were significantly different across the three groups. However, desire, arousal and pain were not statistically different. Subjectively, women cut after marriage had negative sexual experiences, specifically adverse changes in experiences of desire, arousal, and satisfaction.

**Conclusion**

This study revealed sexual disorders associated with FGM/C existed among married women. Thus, mitigating strategies need to be designed to adequately address psycho-sexual complications to improve women’s general well being.

### 2 A cross-country comparison: trends in the adoption and enforcement of Female Genital Mutilation/Cutting laws relating to reinfibulation and vacation cutting

#### Rebecca Reingold^1^, Ranit Mishori^2^, Kevin Fitzgerald^3^, Samantha Wu^3^, Holly Hedley^4^, Rachel Kuenzi^4^, Laura Malavé-Seda^4^

##### ^1^O’Neill Institute for National and Global Health Law, Georgetown University Law Center, Washington DC, USA; ^2^Department of Family Medicine, Georgetown University School of Medicine, MedStar Health – Family Medicine at Spring Valley, Washington DC, USA; ^3^Pellegrino Center for Clinical Bioethics, Georgetown University Medical Center, Washington DC, USA; ^4^Georgetown University Law Center, Washington DC, USA

###### **Correspondence:** Rebecca Reingold (rr951@law.georgetown.edu)

**Introduction**

The objective of this research was to consider how countries are using laws to regulate the practice of Female Genital Mutilation/Cutting (FGM/C), particularly reinfibulation and vacation cutting, and identify areas where legal ambiguity continues to exist for health practitioners who provide care to women and girls who have undergone or who are at risk of undergoing FGM/C.

**Methods**

Cross-country comparison of the laws that regulate FGM/C generally, and the practices of reinfibulation and vacation cutting, in particular. The research focused on laws in select countries in Africa and Europe, as well as Australia and the United States.

**Results**

**T**he research found a series of regional and sub-regional trends related to the adoption and implementation of FGM/C laws, including a movement towards criminalization, low levels of reporting of vacation cutting, legal uncertainty around reinfibulation, increased regulation of medicalized FGM/C and disagreement around how to manage female genital cosmetic surgery in light of FGM/C laws.

**Conclusion**

The research indicates that trends related to the adoption and enforcement of FGM/C laws, particularly those regulating the practices of reinfibulation and vacation cutting, vary between and within regions, and that a range of legal ambiguities related to FGM/C continue to exist for health practitioners around the world.

### 3 Women speak out: Female Genital Cutting, qualitative research

#### Camille Clare^1^, Jacqueline Greenfield^1^, Praise Augustus^1^, Nneamaka Ukatu^1^, Eugene Manu^2^, Brian Altonen^3^

##### ^a^New York Medical College, New York, New York, USA; ^b^Lehman College, Bronx, New York, USA; ^c^New York City Health and Hospitals Corporation, New York, New York, USA

###### **Correspondence:** Camille Clare (camille_clare@nymc.edu)

**Introduction**

Women who have undergone Female Genital Cutting (FGC) may have had varied experiences. This proposed ethnographic, naturalistic inquiry seeks to elucidate the lived experiences of women with FGM/C currently living in the United States. It also examines how women view FGC and the various ways in which the practice has impacted their lives.

**Methods**

Women with FGM/C were identified by the co-principal investigators at the Metropolitan Hospital Center Department of Obstetrics and Gynecology. A semi-structured interview was conducted using standardized and open-ended questions in English. A telephone interpreter service called CYRACOM was utilized for non-English speaking patients. Qualitative data analysis utilized the Health Belief Model and Social Learning or Social Cognitive Theory methodology.

**Results**

Twenty-six women out of 50 identified with FGC agreed to be interviewed. The impact of their experiences was greatest on their sexual activity and spousal relationships. Seventy-two percent of patients reported that being cut affected their sexual relationships in multiple ways including “lack of desire,” “pain,” “lack of pleasure,” and “no feeling during intercourse.” Sixty percent explained that historical, cultural and social pressures influenced having been cut. Thirty percent cited social/personal pressures, as well as an attempt to reduce sexual desire and promiscuity as important contributing factors to FGC. The age at which the patient was cut, family expectations and pressures (from mothers, grandmothers, elder women) and adherence to traditions by the socio-cultural group also contributed to their FGC experience.

**Conclusion**

By understanding the quality and meaning of the patients’ lived experiences with FGC, programs could be developed for health care providers that might lead to their better understanding of the womens’ needs.

### 4 A Belgian multi-disciplinary Female Genital Mutilation medical reference center: a descriptive report of three years of practice

#### Martin Caillet, Fabienne Richard

##### CeMAViE, University Saint Pierre hospital, Brussels, Belgium

###### **Correspondence:** Martin Caillet (Martin_CAILLET@stpierre-bru.be)

**Introduction**

The University Saint Pierre Hospital Medical Center for Assistance to Victims of Excision (CeMAViE) is a public institution located in Brussels, Belgium. Due to its social vocation and location, USPH/CeMAViE welcomes the most people from countries where female genital mutilation/cutting (FGM/C) is practiced. To meet the increasing demand for care, CeMAViE opened its doors in April 2014. CeMAViE is one of the two Belgian reference centers for women with genital mutilations/cutting. In our country, the medical care of women with FGM/C is mandatorily performed only in reference centers approved by the government.

**Methods**

A team consisting of a midwife, a psychologist, a sexologist and a gynaecologist offer multi-disciplinary care and support to the women. Our psychologist uses the Eye Movement Desensitisation and Reprocessing method, a cognitive behavioural therapy. Psychological and sexological care is the first line of treatment. We also perform reconstructive surgery of the clitoris when needed.

**Results**

In three years, we met 667 women during 2000 consultations. Sixty-two surgical procedures were performed, including 29 clitoral reconstructions. The other 33 surgeries were defibulations, excision of cysts and drainage of vulvar abcesses. The majority of our population is between 20 and 39 years old. Their countries of origin are mainly Guinea (51%), Somalia (17%) and Djibouti (11%). They are mainly referred by refugee centers, NGOs and medical doctors. Women come to CeMAVie to consult for medical certificates and to ask questions about reconstruction and gynecological pathology. The feedback from our patients and partners is very positive, which encourages us to continue.

**Conclusion**

Multi-disciplinary care seems to be a good approach to evaluate and treat women with FGM/C. We agree that reference centers must depend on the approval of health authorities. Evidence-based data are now needed to confirm our preliminary impressions: a mixed-methods study, both qualitative and quantitative, will start in the second semester of 2017.

### 5 The pseudo-Clitoris, a particular Female Genital Mutilation: case series

#### Pierre Foldès (pifoldes@gmail.com)

##### Institut en Santé Génésique, Saint Germain en Laye, Paris, France

**Introduction**

The “pseudo-clitoris” is a consequence of a particular way of cutting the clitoris in some countries, such as in Guinea. This FGM is unknown among many healthcare professionals. I present a series of 120 cases of “pseudoclitoris”.

**Methods**

Analysis of 5500 women with FGM who underwent clitoral reconstruction according to Foldès technique. All women attended for reconstructive surgery and underwent local examination and contact ultrasound of the clitoris. All selected women underwent reconstructive surgery and were followed up from 6 to 18 months post surgery.

**Results**

Pseudo-clitoris is a particular type of FGM that was found in 120 out of 5500 cases. The clinical vulvar preoperative anatomy seems normal, but the women report clitoral pain. They often face denial of their story and condition, as the clinical appearance seems normal. There is a single scar above the hood of the glans. The clitoral glans and the labia look normal, but the clitoris has been probably injured by the FGM/C. Reconstructive clitoral surgery according to the Foldès technique has improved sexual function in 76% and pain in 87% of the 120 women.

**Conclusion**

The “Pseudo-Clitoris” must be recognized by experts and professionals who counsel and treat women with FGM/C. It must be considered a real excision/cutting of the clitoris, and managed accordingly.

## VI. Knowledge, Evidence and Consensus Gaps

### 1 Integrative sexual management of Female Genital Mutilation: "Mind Body" proposals. Literature review

#### Béatrice Cuzin^1^, Florence Brunel Delmas^1^, Albertine Papingui^2^

##### ^1^GH E Herriot, Lyon, France; ^2^GAMS, Lyon, France

###### Béatrice Cuzin (beatrice.cuzin@chu-lyon.fr)

**Introduction**

Sexual management of Female Genital Mutilation (FGM) is an under- researched and neglected issue. Available data reported that symptoms, mainly pain and reduced sexual satisfaction and desire, sometimes could be improved by reconstructive clitoral surgery (RCS). Posttraumatic stress disorder (PTSD) is also frequent among women with FGM/C and psychosexual therapies (PST) should be proposed.

**Methods**

The available literature on Sexual Dysfunction (SD) after FGM was identified by searching the PubMed and Cochrane databases from January 1990 to Dec 31, 2016. Search terms related to FGM, SD, RCS, PTSD, PST were used in various combinations.

**Results**

14 studies were found using both terms, FGM and PTSD; only one described results of psychotherapy using eye movement desensitization and reprocessing (EMDR), 6 surgical teams published limited series about RCS effect on sexual improvement with few details on postoperative rehabilitation protocols. No PST study on FGM was found. Looking only at PTSD management, evidence-based therapies are cognitive behavioural therapy (CBT) and EMDR. Mindfulness and osteopathic treatment could be also considered areas of interest to be studied as psychosexual therapies after FGM.

**Conclusion**

In FGM, coping styles of women and PTSD should be considered. An integrative psychosexual therapy, including RCS, CBT, osteopathic treatment and EMDR might be relevant research areas. Research could also be conducted on RCS postoperative rehabilitation protocols.

### 2 Comparative study of sexual function in women post clitoral reconstruction for Female Genital Mutilation/Cutting (FGM/C) and women without FGM/C

#### Sophie Wylomanski^1^, Mathilde Vital ^1^, Sophie De Visme ^2^, Stéphanie Dugast ^1^, Matthieu Hanf ^2^, Norbert Winer ^1^

##### ^1^Department of Gynecology and Obstetrics, Nantes University Hospital, Nantes, France; ^2^ INSERM CIC 1413, Nantes University Hospital, Nantes, France

###### **Correspondence:** Sophie Wylomanski (sophie.wylomanski@gmail.com)

**Introduction**

The aim of this study was to compare sexual function scores in women who had undergone clitoral reconstruction for Female Genital Mutilation/Cutting (FGM/C) to the sexual function scores of women without FGM/C.

**Methods**

Women with FGM/C who underwent clitoral reconstruction at the Nantes University Hospital between 2008 and 2014 were interviewed at least six month after the surgery. They completed a questionnaire describing their sociodemographic and FGM/C characteristics as well as the female Sexual Function Index (FSFI). Each woman with FGM/was matched with three women without FGM/C of the same age.

**Results**

On the 82 women having had clitoral reconstruction, 34 were included. Of them, 23 (68%) had FGM/C Type II. The median summary FSFI score was 29.8 for women with clitoral reconstruction versus 28.8 in the control group. After adjustment, the summary FSFI score was shown to be significantly higher for women with clitoral reconstruction (p.value = 0.05). The same result was observed for desire, satisfaction and pain FSFI subscores.

**Conclusion**

Sexual function scores of women with FGM/C after clitoral reconstruction appeared to be comparable to the scores of a sample of women without FGM/C.

### 3 FGM alerts and expert assessments from healthcare providers: legal case analysis

#### Sara Johnsdotter^1^, Birgitta Essén^2^

##### ^a^ Malmö University, Malmö, Sweden; ^b^ Uppsala University, Uppsala, Sweden

###### **Correspondence:** Sara Johnsdotter (sara.johnsdotter@mah.se)

**Introduction**

Sweden legislated against FGM in 1982. Since then, nearly 90 suspected cases have reached the police and prosecutor. A few of the reports were alerts from healthcare professionals, but the bulk of them originated from daycare and social services sectors. Healthcare providers play a prominent role as experts in forensic investigations.

**Methods**

Cases of suspected FGM originating from healthcare providers who have reported suspected, performed, or planned FGM were analylzed to determine the role of healthcare providers as experts in assessing whether FGM has been performed, and, if so, to what extent.

**Results**

Very few cases analyzed had sufficient indictable evidence; two cases during 35 years were brought to court. The review revealed inconsistencies in the medical assessment processes during which medical experts reached divergent conclusions about FGM status.

**Conclusion**

Variations in normal anatomy and also in cutting procedures make genital assessments by healthcare providers very difficult. It is of utmost importance that appropriate medical experts are summoned in FGM criminal investigations, since these processes often involve radical measures from the police and prosecutor, such as detention of legal custodians and compulsory medical genital examinations of young girls in order to obtain a legally valid medical certificate for an eventual court proceeding.

### **4 Genital reconstructive surgery after female genital mutilation**: **a pre-post study**

#### Amr Seifeldin (amr@seifeldin.org)

##### Urogynecology & pelvic reconstructive surgery unit, El Galaa teaching hospital, Cairo, Egypt

**Background**

Female genital mutilation (FGM), is a cultural tradition widely practiced in Africa and other parts of the world. It can cause serious complications to women’s physical and psychological health. Increased global awareness of the long term consequences of FGM has increased the demand for restorative procedures, yet few doctors are trained in methods of genital cosmetic & reconstructive surgery. Women with FGM/C may be unaware of the availability of clitoral reconstructive surgery to reverse the adverse effects of FGM.

**Method**

One hundred seventeen women with FGM Type II and III between the ages of 18 and 36 years old were selected. They presented to the urogynecology unit at El Galaa Teaching Hospital in Cairo Egypt. Patients answered a female sexual function index (FSFI) questionnaire on admission, noting their sexual characteristics, and pain level. Postoperatively, patients were asked to return for follow up every three months for one year.

**Results**

Clitoral reconstructive surgery after female genital mutilation provided an improvement in the women’s psychology and mood, reflected by an increase in confidence, self-esteem, and body image by 82%. We noted improvement in sexual desire in 24%, arousal in 27% and satisfaction in 16% with moderate improvement in pain reduction (12%), ability to achieve orgasm (8%), and lubrication (4%). Four percent of women were not satisfied with the surgical outcome.

**Conclusion**

Increased education, awareness, and family support are an important step in lowering FGM rates in Africa. Genital reconstructive procedures have shown promising results and should be offered and made more widely available to women with FGM/C who consult gynecology clinics in hospitals. The training of more doctors in genital cosmetic and reconstructive techniques should also be encouraged.

### 5 Making an ethical decision in the exam room: a brief review of the legal, ethical and moral aspects of the clinical management of FGM/C. Case reviews

#### Ranit Mishori^1^, Kevin Fitzgerald^2^, Rebecca Reingold^3^, Samantha Wu^2^

##### ^1^Department of Family Medicine, Georgetown University School of Medicine, MedStar Health – Family Medicine at Spring Valley, Washington DC, USA; ^2^ Pellegrino Center for Clinical Bioethics, Georgetown University Medical Center, Washington DC, USA; ^3^O’Neill Institute for National and Global Health Law, Georgetown University Law Center, Washington DC, USA

###### **Correspondence:** Ranit Mishori ^(^mishorir@georgetown.edu)

**Introduction**

Clinicians who work with women who with FGM/C can face a clash of medical, moral, ethical and legal obligations surrounding professional duty, obligations to the patient, respect for her autonomy and culture, and regard for local laws, regulations, policies and human rights. Conflicts arise whether local laws exist or not: What should a clinician do when asked to reinfibulate? How is FGM/C or reinfibulation different from labiaplasty? Are there potential harms to reporting ‘vacation cutting’? Is performing FGM/C in a health facility an ethically legitimate means of harm reduction’?

**Methods**

We briefly review six cases highlighting real-life ethical and legal conflicts and propose an ethical decision making framework and an affiliated online and smart phone App to use to help guide clinical and professional decisions. There are 6 simulated cases that illustrate real-life situations.

**Results**

Cases featured include vacation cutting, reinfibulation after delivery, the practice of vaginoplasty, the medicalization of FGM/C, reporting on colleagues who are believed to engage in FGM/C, and considering alternative FGM/C practices.

**Conclusion**

This is an educational intervention using an app, and it has not yet been evaluated. It is an exercise in ethical decision making for residents and physicians to consider the various approaches and options for action, to discuss and reflect on how their individual decisions can be implemented with the greatest care and attention to the concerns of all stakeholders.

### 6 Reparative approaches: the different meanings of « reparation » for women living with FGM in France and Switzerland. Qualitative Analysis

#### Michela Villani (michela.villani@unifr.ch)

##### University of Fribourg, Fribourg, Switzerland

**Introduction**

Based on two qualitative studies, we aim to explore the sexual health of women living with FGM in France and in Switzerland.

**Methods**

We conducted semi-structured, in-depth interviews with a group of 8 immigrant women of sub-Saharan origin living in Switzerland with Type III FGM (infibulation) and 32 women of first and second generation living in France with Type II FGM (excision). All of the participants had either had or asked for a clitoris reconstruction. All women described their own perceptions of health, reproductive life and sexuality.

**Results**

The group of women with infibulation and the group of women with excision differ in their socio-demographic characteristics and the context of FGM. Both groups affirmed their desire to improve, or at least change, their conditions. Reparative approaches are sought by women in order to « repair » something « lost »; the word « reparation » acquires a large scale of meanings. The first type of request relates to the physical reparation, expressed by the women’s feeling of “having been damaged”. Excised/infibulated women living in the North say they feel “dissatisfied” or “unhappy” with their sexual experiences. The feeling that something has been “lost” or “stolen” seems to produce the desire to seek justice. The reconstructed clitoris then may be a perceived as a material symbol of a kind of reward for endured suffering - that I call here the “moral reparation” (or symbolic reparation). Gender also plays an important role in terms of body image and gender models that differ from those of previous generations of women in their family.

**Conclusion**

In this study, women chose reparative approaches for a number of reasons. Specific socio-sexual management is recommended when caring for immigrant women living with FGM in order to respond to their specific health care needs. Multidisciplinary approaches may be able to offer more comprehensive health care, in order to improve dialogue.

### 7 The increasing demand for reconstructive clitoral surgery among circumcised women living in Europe. A nexus analysis

#### Sara Johnsdotter^1^, Birgitta Essén^2^

##### ^1^Malmö University, Malmö, Sweden; ^2^ Uppsala University, Uppsala, Sweden

###### **Correspondence:** Sara Johnsdotter (sara.johnsdotter@mah.se)

**Introduction**

The demand for the surgical technique of clitoral reconstructive surgery introduced by Pierre Foldès is increasing globally despite lack of evidence of its benefits weighed against its potential harms.

**Methods**

In an ongoing study, we use nexus analysis to study the introduction of the surgery in Sweden. In nexus analysis, one simultaneously reviews current discourses, actors and settings to understand a particular phenomenon. In this analysis we seek to determine who is promoting the surgery, in what settings, and what discourses are offered to interpret the phenomenon.

**Results**

Preliminary results, in line with other studies from social science literature, suggest that reconstructive clitoral surgery as a biomedical practice is a response to Western discourses on ‘female genital mutilation’: discourses that label cut women as ‘mutilated’, sexually deprived and less feminine than uncut women. These discourses in themselves are harmful to women and may cause them to seek a surgical solution that may not actually lead to improved outcomes.

**Conclusion**

A new biomedical surgical procedure has been introduced and is generally praised, despite the lack of evidence to prove beneficial outcomes. This surgery is embedded in a powerful discourse that may negatively affect far more women than those who opt for surgery. The negative effects of female genital cutting should be carefully addressed in campaigning in order not to stigmatize already cut women further.

### 8 Could efforts to eliminate female genital cutting be strengthened by extending protections to male and intersex children too? A commentary

#### Rebecca Seinfeld^1^, Brian Earp^2^

##### ^1^Visiting Research Fellow, Centre of the Body, Goldsmiths, University of London, London, United Kingdom; ^2^Associate Director, Yale-Hastings Program in Ethics & Health Policy and Departments of Philosophy and Psychology, Yale University, New Heaven, Connecticut, USA

###### **Correspondence:** Rebecca Seinfeld (rebecca@rebeccasteinfeld.com)

**Introduction**

Activists and academics disagree about the most effective ways to eliminate non-therapeutic female genital cutting (FGC). Some argue that efforts to reduce FGC are undermined by conflations of the least and most invasive types, unsubstantiated claims about universally negative effects, and inflammatory language. Scholars also argue that failing to apply the principles of bodily integrity to children of all sexes will ultimately backfire due to (a) incompatibility with equality principles, and (b) recent calls by defenders of male circumcision to permit ritual nicking of females for intellectual consistency.

**Methods**

Drawing on ethical critiques and anthropological studies, we ask: Would the campaign against FGC be strengthened or weakened by including male and intersex children?

**Results**

We find that a gender-inclusive approach would: (1) neutralize accusations of cultural imperialism by applying the same standards to white children in the USA as to children of color in Africa; (2) weaken accusations of sexism by recognizing that boys and intersex children are also vulnerable to non-therapeutic genital alteration; (3) redress the moral confusion in communities that practice both female and male genital-alterations caused by Western attempts to eliminate only the female “half” of their initiation rites.

**Conclusion**

We find that efforts to eliminate FGC will be more successful if they expand to include vulnerable persons of all genders.

## VII. Abstracts presenting already published papers

### 1 Female genital mutilation: Knowledge, attitude and practices of Flemish midwives (Belgium)

#### Cappon S^1^, L'Ecluse C^1^, Clays E^2^, Tency I^3^, Leye E^1^

##### ^1^Faculty of Medicine, International Centre for Reproductive Health, Ghent University. Gent, Belgium; ^2^ Department Public Health, Ghent University, Gent, Belgium; ^3^Midwifery Department, KAHO Sint Lieven, Sint Niklaas, Belgium

###### **Correspondence:** Leye E (els.leye@ugent.be)

**This abstract was previously published as**

Cappon S, L'Ecluse C, Clays E, Tency I, Leye E. Female genital mutilation: knowledge, attitude and pratices of Flemish midwives. Midwifery. 2015 Mar;31(3):e29-35. doi: 10.1016/j.midw.2014.11.012

### 2 Cultural values affecting the acceptance of surgical defibulations

#### Johansen RE^1^ (johansen@nkvts.no)

##### ^1^Norwegian Center for Violence and Traumatic Stress Studies, Oslo, Norway.

**This abstract was previously published as**

Johansen RE. Virility, pleasure and female genital mutilation/cutting. A qualitative study of perceptions and experiences of medicalized defibulation among Somali and Sudanese migrants in Norway. Reprod Health. 2017 Feb 10;14(1):25. doi: 10.1186/s12978-017-0287-4.

Johansen RE. Undoing female genital cutting: perceptions and experiences of infibulation, defibulation and virginity among Somali and Sudanese migrants in Norway. Cult Health Sex. 2017 Apr;19(4):528-542. doi: 10.1080/13691058.2016.1239838.

### 3 Clitoral reconstruction at CHU Yalgado of Ouagadougou, Burkina Faso

#### Ouédraogo CM^1^, Madzou S^2^, Simporé A^1^, Combaud V^2^, Ouattara A^1^, Millogo F^1^, Ouédraogo A^1^, Kiemtore S^1^, Zamane H^1^, Sawadogo YA^1^, Kaien P^1^, Dramé B^1^, Thieba B^1^, Lankoandé J^1^, Descamps P^2^

##### ^1^Division of Gynecology and Obstetrics, CHU Yalgado Ouédraogo, Ouagadougou, Burkina Faso; ^2^Pôle Femme-Mère-Enfant, CHU d'Angers, Angers, France

###### **Correspondence:** Ouédraogo CM (ocharlemagne@yahoo.fr)

**This abstract was previously published as**

Ouédraogo CM, Madzou S, Simporé A, Combaud V, Ouattara A, Millogo F, Ouédraogo A, Kiemtore S, Zamane H, Sawadogo YA, Kaien P, Dramé B, Thieba B, Lankoandé J, Descamps P. Clitoral reconstruction after female genital mutilation at CHU Yalgado of Ouagadougou, Burkina Faso. About 68 patients operated]. J Gynecol Obstet Biol Reprod (Paris). 2016 Nov;45(9):1099-1106

### 4 Male perspectives on FGM among communities of African heritage in Italy

#### Catania L^1^, Mastrullo R^1^, Caselli A^1^, Cecere R^1^, Abdulcadir O^1^, Abdulcadir J^2,3^

##### ^1^Regional Referral Centre for the Treatment and Prevention of FGM, Health Promotion of Immigrant Woman, Department of Science for Woman and Child Health, University of Florence, Florence, Italy; ^2^Faculty of Medicine, University of Geneva, Geneva, Switzerland; ^3^Department of Obstetrics and Gynecology, Geneva University Hospitals, Geneva, Switzerland

###### **Correspondence:** Abdulcadir O (oabdulcadir@gmail.com)

**This abstract was previously published as**

Catania L, Mastrullo R, Caselli A, Cecere R, Abdulcadir O, Abdulcadir J^3^. Male perspectives on FGM among communities of African heritage in Italy, International Journal of Human Rights in Healthcare, Vol. 9 Iss: 1, pp.41 – 51. DOI http://dx.doi.org/10.1108/IJHRH-07-2015-0023

### 5 Changing cultural attitudes on FGC: Experimental randomized trial

#### Sonja Vogt, Charles Efferson

##### University of Zurich, Zurich, Switzerland

###### **Correspondence:** Sonja Vogt (sonja.vogt@econ.uzh.ch)

**This abstract was previously published as**

Efferson C, Vogt S, Elhadi A, El Fadil Ahmed H, Fehr E. Female genital cutting is not a social coordination norm. Science 25 Sep 2015: Vol. 349, Issue 6255, pp. 1446-1447 DOI: 10.1126/science.aaa7978.

Vogt S, Ahmed Mohamed Zaid N, El Fadil Ahmed H, Fehr E, Efferson C. Changing cultural attitudes towards female genital cutting. Nature, 538, 506–509 (27 October 2016) doi:10.1038/nature20100

### 6 Men have a role to play but they don’t play it. A mixed methods study exploring men’s involvement in Female Genital Mutilation in Belgium, the Netherlands and the UK

#### O’Neill S^1^, Dubour D^2^, Florquin S^2^, Bos M^3^, Zewolde S^4^, Richard F^1^

##### ^1^Institute of Tropical Medicine, Antwerp. Antwerp. Belgium; ^2^ GAMS, Brussels, Belgium; ^3^ HIMILO Foundation. Amsterdam. The Netherlands; ^4^ FORWARD UK. London. United Kingdom

###### **Correspondence:** O’Neill S (soneill@itg.be)

**This abstract was previously published as**

O’Neill S, Dubour D, Florquin S, Bos M, Zewolde S, Richard F. “Men have a role to play but they don’t play it”: A mixed methods study exploring men’s involvement in Female Genital Mutilation in Belgium, the Netherlands and the UK. Summary. Men Speak Out Project, Brussels, 2017.

### 7 **Obstetric outcomes for women with female genital mutilation at an Australian hospital, 2006-2012: a descriptive study**

#### Varol N^1*^, Dawson A^2^, Turkmani S^2^, Hall JJ^3^, Nanayakkara S^4^, Jenkins G^4,5^, Homer CS^2^, McGeechan K^6^.

##### ^1^ Discipline of Obstetrics and Gynaecology, Sydney Medical School, University of Sydney, Sydney, NSW, Australia; ^2^ Centre for Midwifery, Child and Family Health, Faculty of Health, University of Technology Sydney, Sydney, NSW, Australia; ^3^ Centre for Clinical Epidemiology & Biostatistics, School of Medicine and Public Health, Faculty of Health and Medicine, University of Newcastle, Sydney, NSW, Australia; ^4^ Department of Obstetrics and Gynaecology, Auburn Hospital, Sydney, NSW, Australia; ^5^ Discipline of Obstetrics and Gynaecology, School of Medicine, Notre Dame University, Sydney, NSW, Australia; ^6^ School of Public Health, University of Sydney, Sydney, NSW, Australia.

###### **Correspondence:** Varol N (nesrin.varol@sydney.edu.au)

**This abstract was previously published as**

Varol N, Dawson A, Turkmani S, Hall JJ, Nanayakkara S, Jenkins G, Homer CS, McGeechan K. Obstetric outcomes for women with female genital mutilation at an Australian hospital, 2006-2012: a descriptive study. BMC Pregnancy Childbirth. 2016 Oct 28;16(1):328.

### 8 Using the Female Sexual Function Index (FSFI) to evaluate sexual function in women with genital mutilation undergoing surgical reconstruction: a pilot prospective study

#### Vital M^1^, de Visme S^2^, Hanf M^2^, Philippe HJ^1^, Winer N^1^, Wylomanski S^3^

##### ^1^ Department of Gynecology and Obstetrics, Nantes University Hospital, Nantes, France; ^2^ INSERM CIC 1413, Nantes University Hospital, Nantes, France; ^3^ Department of Gynecology and Obstetrics, Nantes University Hospital, Nantes, France.

###### **Correspondence:** Wylomanski S (sophie.wylomanski@gmail.com)

**This abstract was previously published as**

Vital M, de Visme S, Hanf M, Philippe HJ, Winer N, Wylomanski S. Using the Female Sexual Function Index (FSFI) to evaluate sexual function in women with genital mutilation undergoing surgical reconstruction: a pilot prospective study. Eur J Obstet Gynecol Reprod Biol. 2016 Jul;202:71-4. doi: 10.1016/j.ejogrb.2016.04.029

### 9 Interventions to Address Sexual Function in Women Affected by Female Genital Cutting: a Scoping Review

#### Johnson-Agbakwu C^1,2^, Warren N^3^

##### ^1^Founding Director, Refugee Women’s Health Clinic, Maricopa Integrated Health System, Obstetrics & Gynecology, Phoenix, Arizona, USA; ^2^Assistant Research Professor, Arizona State University, Southwest Interdisciplinary Research Center, Phoenix, Arizona, USA; ^3^ Department Cmmunity Public Health Nursing. Johns Hopkins School of Nursing. Baltimore, Maryland, USA

###### **Correspondence:** Warren N (nwarren3@jhu.edu)

**This abstract was previously published as**

Johnson-Agbakwu C, Warren N. Interventions to Address Sexual Function in Women Affected by Female Genital Cutting: a Scoping Review. Curr Sex Health Rep (2017) 9: 20. doi:10.1007/s11930-017-0099-0

### 10 Estimation of the prevalence of female genital mutilation/cutting among migrant women living in England and Wales

#### Macfarlane A^1^, Dorkenoo W^1^

##### ^1^ City, University of London. London. United Kindgom.

###### **Correspondence:** Macfarlane A (a.j.macfarlane@city.ac.uk)

**This abstract was previously published as**

Macfarlane A, Dorkenoo W. Prevalence of Female Genital Mutilation in England and Wales: National and local estimates. Published by City University London, Northampton Square, London EC1V 0HB and Equality Now, 1 Birdcage Walk, London SWIH 9JJ July 2015 ISBN 978-1-900804-93-6.

http://openaccess.city.ac.uk/12382/

### **11 The role of medical doctors and the role of the Child Protection Services in detecting cases of female genital mutilation within the system of law in Norway**: l**egal case reviews**

#### Lien IL^1^, Schultz JH^1^

##### ^1^ Norwegian Centre for Violence and Traumatic Stress Studies, Oslo, Norway.

###### **Correspondence:** Lien IL (i.l.lien@nkvts.no)

**This abstract was previously published as**

Lien IL.Prosecution of the Offence of Female Genital Mutilation/Cutting in Norway. Int J Law Policy Family 2017 ebx003. doi: 10.1093/lawfam/ebx003

Lien IL, Schultz JH. Interpreting Signs of Female Genital Mutilation Within a Risky Legal Framework. Int J Law Policy Family, 2014, 28(2): 194–211 doi: 10.1093/lawfam/ebu002

